# Green Protocols in Heterocycle Syntheses via 1,3-Dipolar Cycloadditions

**DOI:** 10.3389/fchem.2019.00095

**Published:** 2019-02-26

**Authors:** Katia Martina, Silvia Tagliapietra, Valery V. Veselov, Giancarlo Cravotto

**Affiliations:** ^1^Dipartimento di Scienza e Tecnologia del Farmaco and NIS, Centre for Nanostructured Interfaces and Surfaces, University of Turin, Turin, Italy; ^2^Center of Bioanalytical Research and Molecular Design, Institute for Translational Medicine and Biotechnology, First Moscow State Medical University (Sechenov), Moscow, Russia

**Keywords:** green chemistry, dipolar cycloadditions, heterocycles, aqueous medium, Ionic liquids

## Abstract

The aim of this review is to provide an overview of green protocols for the organic synthesis of heterocycles via 1,3-dipolar cycloaddition. Particular attention has been devoted to the use of green solvents; reactions performed in ionic liquids, fluorinated solvents and water have been included. Also explored are several protocols that make use of catalyst-free reaction conditions, the use of microwave irradiation and activation by light exposure. Improvements over commonly used organic solvents will be underlined in order to highlight environmental protection aspects and enhancements in regio- and stereo-selectivity.

## Introduction

The design of efficient and sustainable synthetic protocols is of primary importance to green pharmaceutical chemistry. The selection of suitable starting materials, methodologies with good atomic balance, a minimum number of steps and green solvents will allow this beneficial goal to be achieved. Heterocyclic chemistry plays a key role in drug production and the application of efficient green synthetic processes has a significant impact on pharmaceutical industries. There are a number of successful examples of benign synthetic protocol, including the use of non-conventional technologies such as microwave of ultrasound irradiations (Garella et al., 2013) and the use of water or green organic solvents (Moulay and Touati, 2010; Butler and Coyne, 2016). In the last decade, in this area, green chemistry and click chemistry have respected a pathway of rigorous principles, by means of which more efficient and greener processes can be defined (Anastas and Eghbali, 2010). Since the capital discovery by the teams of Meldal (Tornøe et al., 2002) and Sharpless (Rostovtsev et al., 2002), copper-catalyzed azide-alkyne cycloaddition (CuAAC) provides regioselectivity under mild conditions substituted triazole. Based on its large applicability, several research areas have taken advantage of its peculiar benefits and this reaction can be considered the click reaction *par antonomasia*. More in general, 1,3-dipolar cycloadditions are six π-electrons, concerted reactions that have a wide range of applications in organic synthesis and, specifically, in the preparation of five membered heterocyclic rings. Their good atomic balance and their synthetic potential resulted maximized when 5 terms heterocycles are obtained in sustainable solvents with high stereo or enantioselectivity.

Many authors still use the terms “[2+3] or [3+2] cycloaddition,” which count the number of involved atoms, but do not follow IUPAC recommendations. In this paper, we follow the IUPAC requirements, meaning that all reactions are referred to in accordance with the following rules:

- *A (i* + *j* +…*) cycloaddition is a reaction in which two or more molecules (or parts of the same molecule), provide units of i, j, in which i and j stand for linearly connected atoms. In the final product, these units become joined by new* σ*-bonds so as to form a cycle containing (i* + *j* +…*) atoms*.
- *The terminology [i* + *j* +…*] for a cycloaddition identifies the numbers, i and j, of π electrons in the interacting units that participate in the transformation of reactants to products* (see http://goldbook.iupac.org/html/C/C01496.html).

The aim of this review is to provide an overview of green protocols for the organic synthesis of heterocycles via 1,3-dipolar cycloaddition. The azides-alkynes cycloadditions, well-known as Huisgen reactions, will not be taken into detailed account because of their large presence in literature, both in original publication and in review. In the present review particular attention has been devoted to the use of green solvents; reactions performed in ionic liquids, fluorinated solvents and water have been included. Also explored are several protocols that make use of catalyst-free reaction conditions, the use of microwave irradiation and activation by light exposure. Improvements over commonly used organic solvents will be underlined in order to highlight environmental protection aspects and enhancements in regio- and stereoselectivity.

## 1,3-Dipolar Cycloaddition in Ionic Liquids

The elimination of the use of volatile organic solvents and their replacement by non-inflammable, non-volatile, non-toxic, and inexpensive green solvents is an important aspect of green chemistry. Their unique properties, including high chemical and thermal stability, solvating ability, ability to behave as both acidic and basic catalysts and recyclability, have led to ionic liquids gaining widespread recognition as green solvents that are advantageous to use in organic synthesis. Their manifold use in this context has therefore emerged as a formidable ally for green chemistry. Besides the catalyzing ability of ionic liquids, their solubility, viscosity, density, acidic, or basic character and refractive index, can be tuned by judiciously modifying the anion/cation combinations. This means that for multicomponent reactions and 1,3-dipolar cycloadditions, they might be the reaction media of choice.

**Scheme 1:**
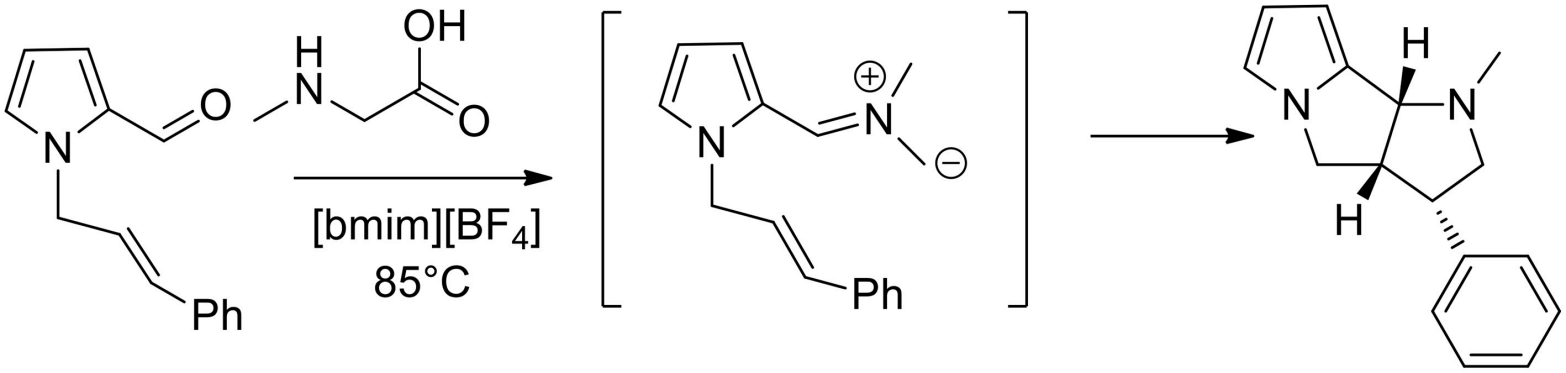
Synthesis of *N*-methylpyrrol[2,3-a]pyrrolizidine.

In 2007, 1,3-dipolar azomethine ylide cycloaddition has been studied in ionic liquid and the authors observed that pyrrolizidines can be obtained in high yield at 50°C in ionic liquid 1-butyl-3-methylimidazolium [bmim][BF_4_] in 10–40 min, while under conventional conditions in boiling acetonitrile it requested 90–300 min (Jadidi et al., 2007). Kathiravan and Raghunathan (2013) studied an intramolecular 1,3-dipolar in [bmim][BF_4_] as the medium and pyrrolo[2,3-a]pyrrolizidino derivatives were obtained, as described in Scheme 1. Starting from pyrrole-2-carbaldehyde and allyl bromide, the authors obtained N-alkenyl pyrrole-2-carbaldehyde that converted in presence of sarcosine in pyrrolo[2,3-a]pyrrolidines, pyrrolizidines, indolizidines, and isoquinolines. The reaction was performed at 85°C and in 3 h, and pyrrolopyridines were obtained in good yields (80–92%) from the 1,3-dipolar cycloaddition reactions. To provide an optimization of the method the authors also carried out the reaction in different organic solvents, including methanol, toluene, and acetonitrile. The reaction was slower and afforded lower yields of the desired products when performed in the organic solvents compared to ionic liquid.

**Scheme 2:**
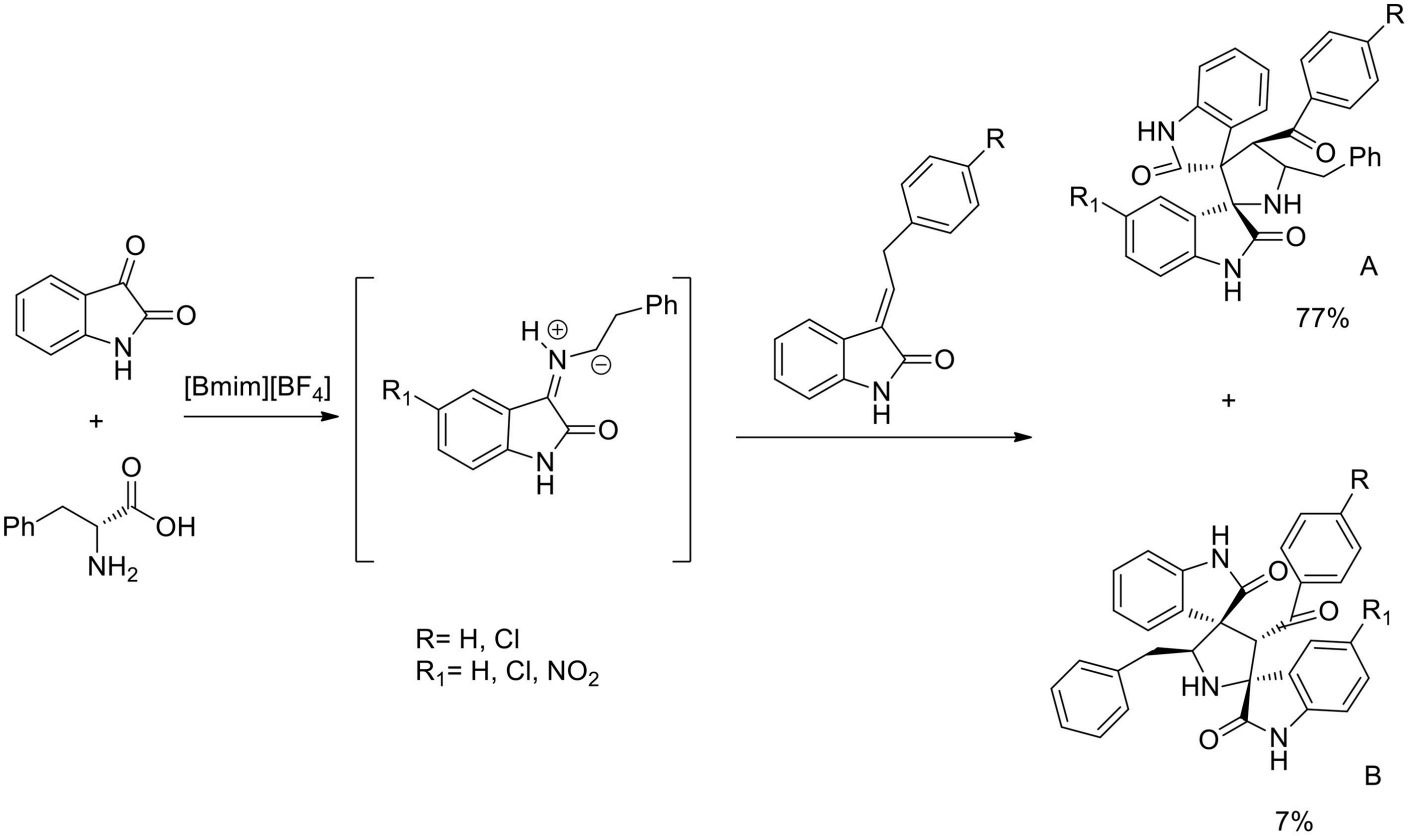
Synthesis of dispirooxindolopyrrolidines.

**Scheme 3:**
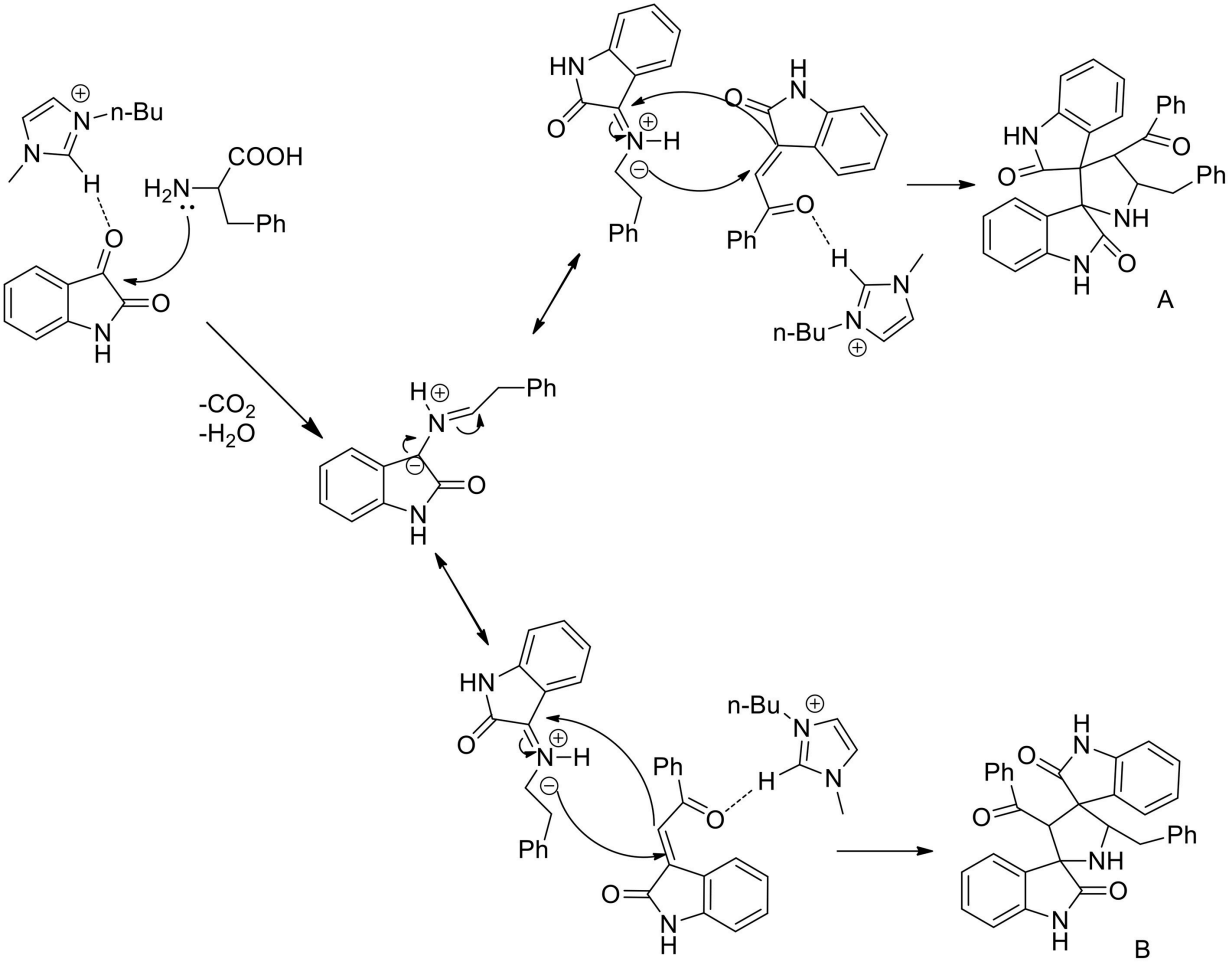
Possible mechanism of the synthesis of dispirooxindolopyrrolidine regioisomers.

A three component 1,3-dipolar cycloaddition, one pot reaction has been described (Almansour et al., 2015) to obtain dispirooxindolopyrrolidines. *In situ* generated azomethine ylides from L-phenylalanine and substituted isatins were used in equimolar ratio with a series of (E)-2-oxoindolino-3-ylidene acetophenone in [bmim][BF_4_] to furnish the cycloadducts in 70–77% yield and high regioselectivity (Scheme 2). When compared with organic solvent such as methanol, ethanol, dioxane, and a dioxane/methanol (1:1) mixture under heating in an oil-bath, they gave the desired product in lower yield (28–38%) with low selectivity. The same reaction was investigated in ionic liquids using catalyst in 10 mol%, [bmim][BF_4_]/CuI, [bmim][BF_4_]/Zn(OTf)_2_. These reactions furnished both regioisomers A and B, but good yield and high selectivity were observed toward the product A. The ionic liquids [bmim]BF_4_ and [bmim]Br were found to be the most suitable reaction media and as described by the authors in the proposed mechanism (Scheme 3), ionic liquids play the dual role of solvent and catalyst and an increase in reaction rate is observed when compared to other organic solvents.

**Scheme 4:**
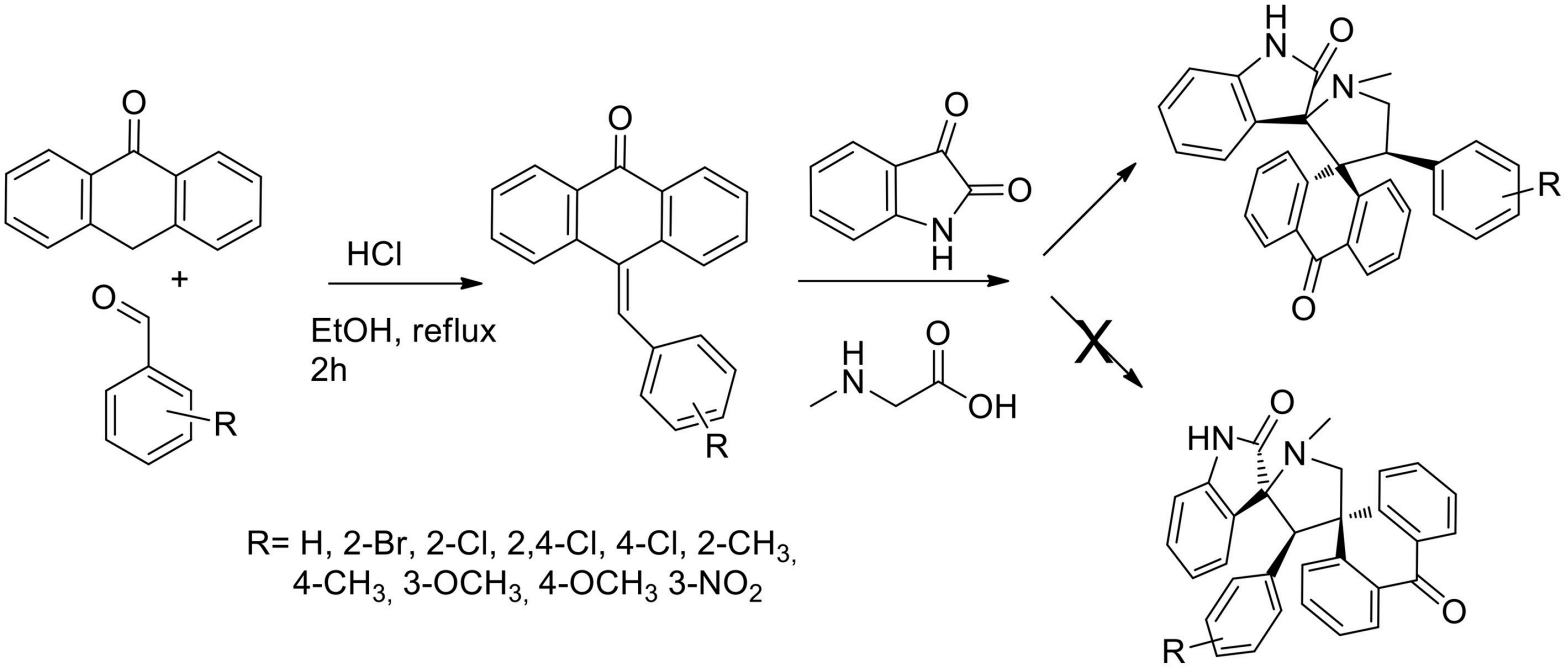
Synthesis of 9-arylmethylene-10-anthrone and following 1,3-dipolar cycloaddition to bis-spiro compound.

Another example of one pot three components reactions was published in 2015 by the same authors (Arumugam et al., 2015) that described a general and efficient path for the regio- and stereo-selective synthesis of dispirooxindole-fused anthrones. The reaction was a reaction pathway (Scheme 4) started from acid-catalyzed condensation of anthracen-9(10*H*)-one in presence of benzaldehydes to obtain 10-benzylideneanthracen-9(10*H*)-ones that was submitted to 1,3-dipolar cycloaddition with non-stabilized azomethine ylide from sarcosine and isatin (*in situ* generated). When performed in ethanol, methanol, dioxane, and a dioxane/methanol (1:1 v/v) at reflux, the 1,3-dipolar cycloaddition failed. The desired product was obtained in 65% yield in 3 h in DMF at reflux. Working in the presence of [bmim]Br at 100°C, the product was obtained in high yield (89%) and the reaction rate increased compared to DMF. Moreover, the ionic liquid was recovered and reused. The reaction was proved as regioselective. The reaction showed high tolerance toward substituted benzaldehyde and electron donating as well as electron-withdrawing substituents at *ortho, meta*, and *para* positions gave satisfactory yields. ^1^H-,^13^C-, and 2D-NMR spectroscopy was exploited to elucidate the stereochemistry of the final product and unambiguously the stereochemistry was confirmed using a single crystal X-Ray diffraction study.

**Scheme 5:**
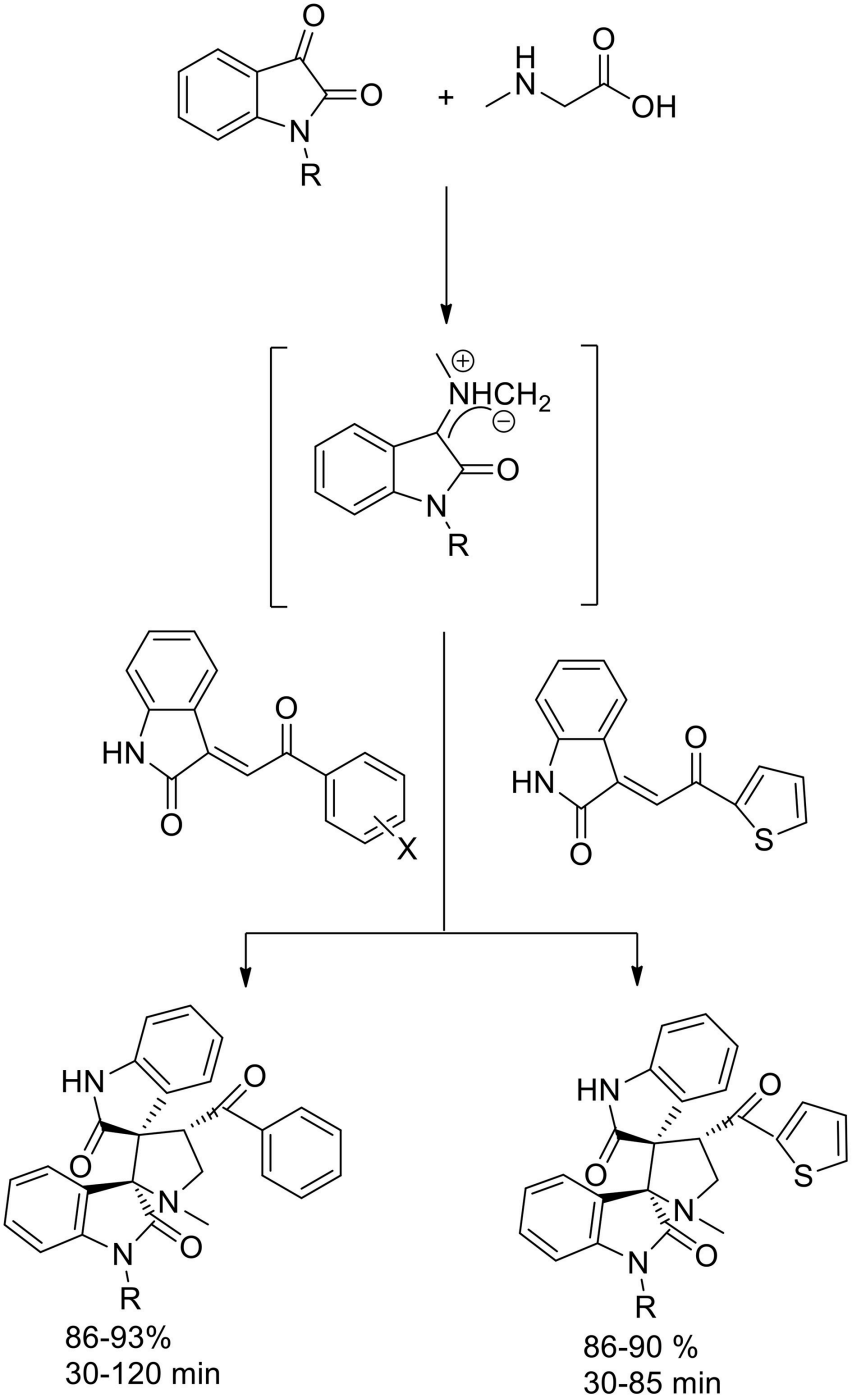
Synthesis of dispiropyrrolidine derivatives.

Also Jain et al. (2012) reported the enhancement in yield and rate due to the use of ionic liquid in 1,3-dipolar cycloaddition reaction *via* the generation of azomethine ylides from isatin and sarcosine (Scheme 5) to synthesize dispiropyrrolidine-bisoxindole. The dipolarophile employed was the olefin obtained by Knoevenagel condensation of isatin and un/substituted acetophenones and 2-acetylthiophene. In presence of [bmim]PF_6_, the azomethine ylides approached the dipolarophiles regioselectively and excellent yields in short reaction times were obtained. Furthermore, there was no need for further purification processes, such as column chromatography, and the products were characterized by spectroscopic and analytical studies.

**Scheme 6:**
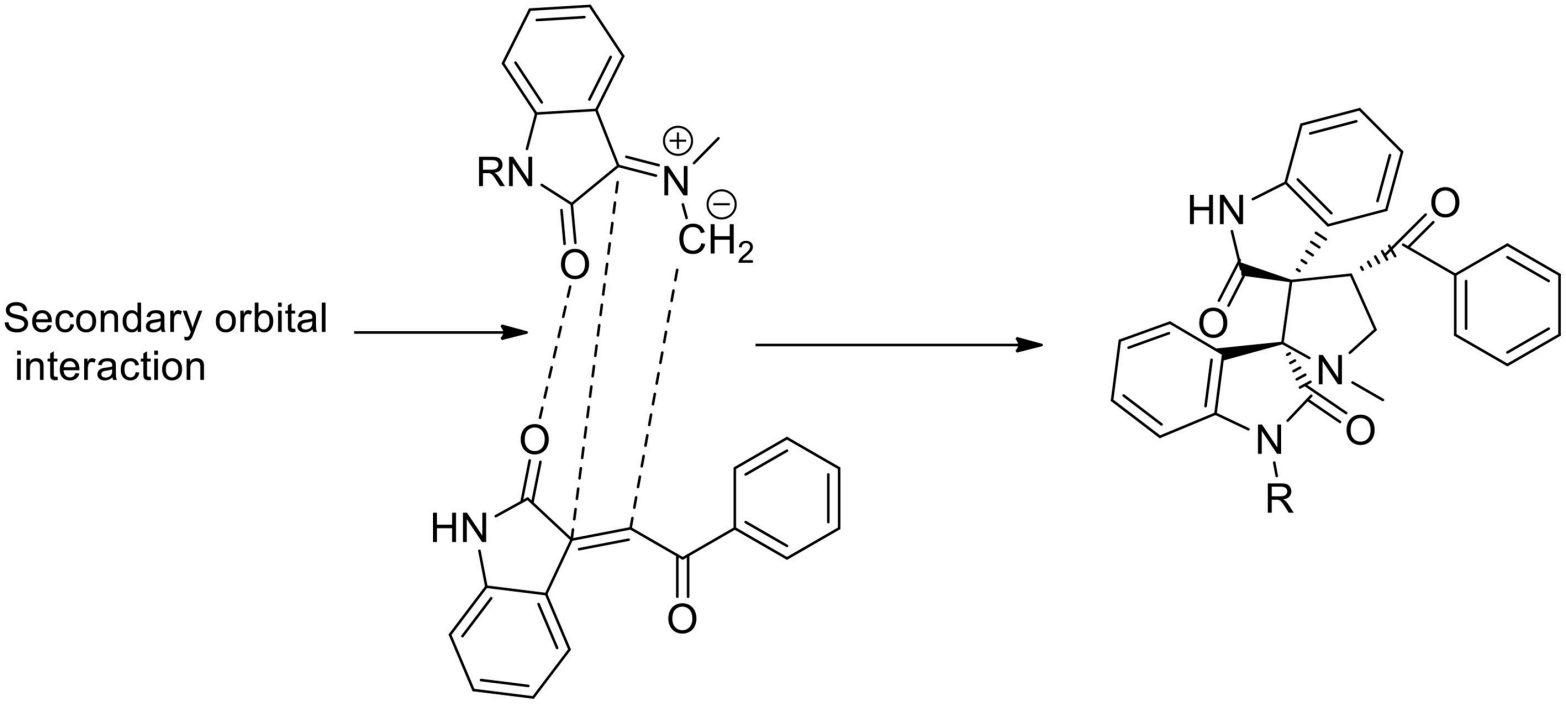
Schematic representation of plausible secondary orbital interaction of azomethine ylide.

The stereoselectivity of the 1,3-dipolar cycloaddition is governed by both the orientation at which the dipole and dipolarophile approached and the conformation of the ylide. In the mentioned study, the authors hypothesize that constraints dictate that only one specific isomer can be involved in the transition state of the cycloaddition reaction. As described in the Scheme 6, the reaction occurs through one of the ylide geometries therefore its addition to dipolarophiles takes place under the control of relative stereochemistry at the spiro center. The formation of sterically hindered ylide was not obtained owing to steric repulsion between the oxindole carbonyl group and the methyl group of sarcosine. Ionic liquid recyclability was proved by the authors.

**Scheme 7:**
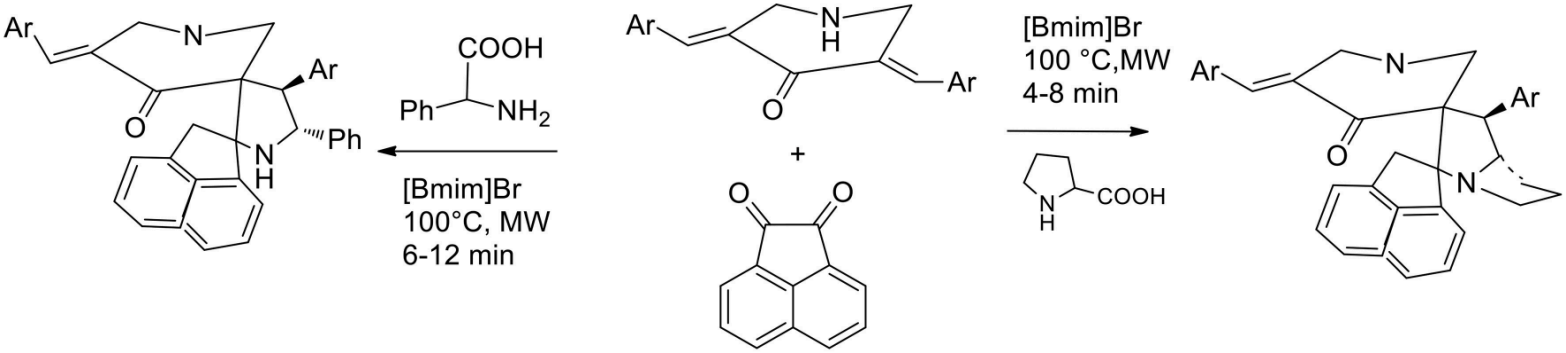
Synthetic route of diazapoycyclic cage compounds.

The parallel synthesis of novel molecular frameworks has been greatly enhanced by the discovery of the synergy between microwave irradiation and ionic liquid. Being a heterocycle of immense importance because of its potential biological activity, 1,3-dipolar cycloaddition under MW irradiation in ionic liquid has been explored for facilitating accelerations in drug discovery. Kumar et al. (2015) have studied the molecular recognition of cage compounds and attempted the incorporation into their structure or a second cavity that can participate in structure recognition. 1,3- dipolar cycloaddition between *N*-unsubstituted 3,5-*bis*[(E)-arylmethylidene]tetrahydro- 4(1H)-pyridinones and azomethine ylides was exploited for the synthesis of novel diazahexa- and diazaheptacyclic ring. Azomethine ylides were generated *in situ* from acenaphthenequinone and α-amino acids (initially, proline and then phenylglycine). Initially the authors performed the reaction under conventional conditions and methanol, ethanol, ethanol−1,4-dioxane mixture (1:1 v/v), and 1,4-dioxane were employed in refluxing to afford the heptacyclic cage structure in 72, 60, 70, and 69% yields, respectively. When the reaction was performed in [bmim]Br at 100°C, the desired product was recovered in 81% yield in 20 min. The synergic effected of MW irradiation and ionic liquid was proved when an equimolar mixture of the starting materials in [bmim]Br was subjected to MW irradiation at 100°C for 4 min. The cage compound was recovered in 85% yield after purification by extraction and crystallization. The ionic liquid [bmim]Br was dried under reduced pressure after product extraction and its recyclability was investigated in successive syntheses of the cage compound, revealing that its efficacy was not particularly reduced in the three subsequent runs (Scheme 7).

**Scheme 8:**
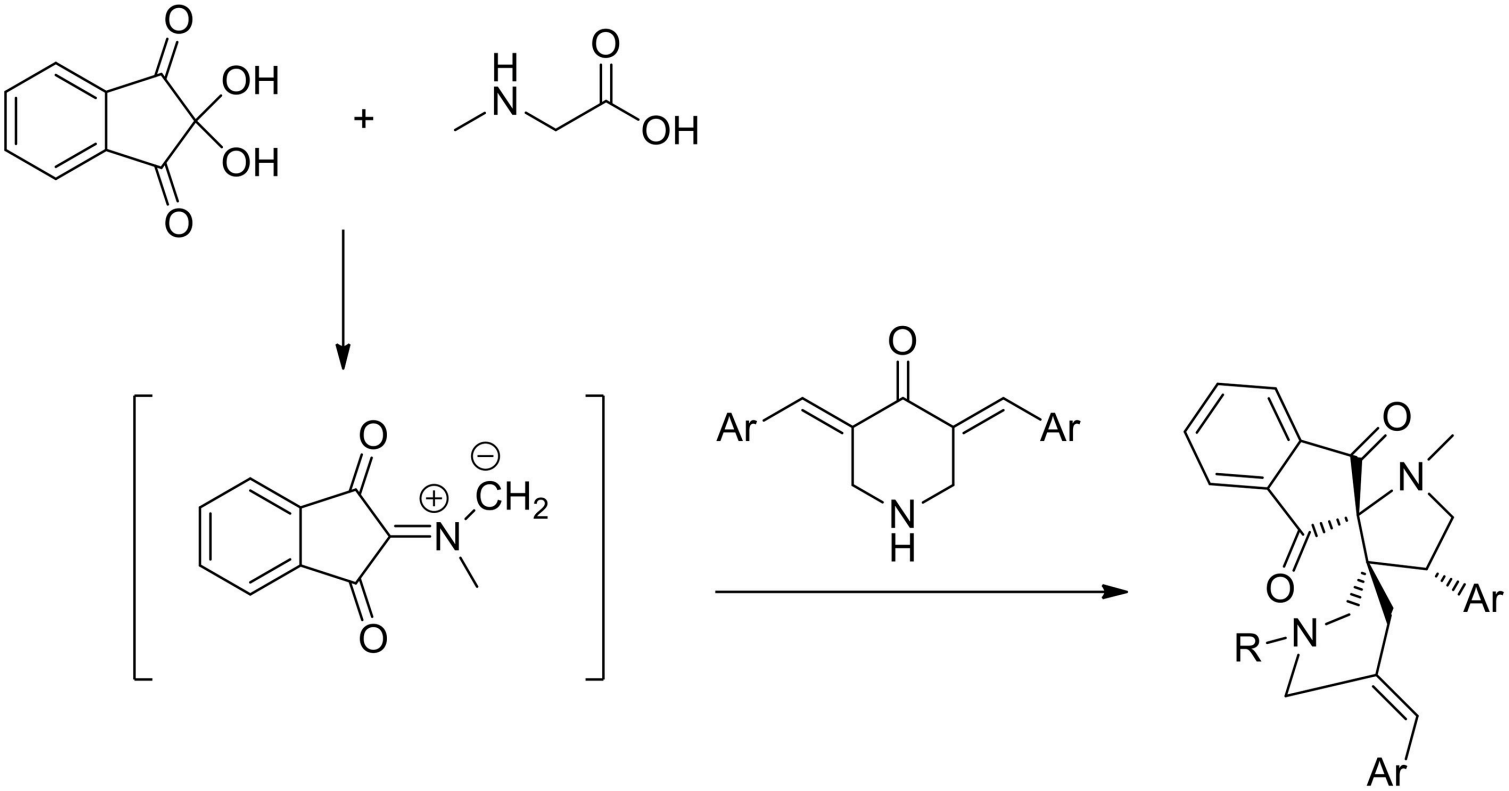
Synthesis of dispiropyrrolidine derivatives.

Recently guanidine ionic liquids (GILs) have also emerged and have been efficiently used, as new-generation ionic liquids, in several reactions (Henry reaction, aldol reaction, Heck reaction etc.). The preparation of novel dispiropyrrolidine derivatives under green conditions was studied by Dandia et al. (2012) exploiting the task-specific ionic liquid 1,1,3,3-tetramethylguanidine acetate [TMG][Ac] as the reusable solvent in a 1,3-dipolar cycloaddition. The reaction was carried out in the presence of an equimolar mixture of sarcosine, ninhydrin, and 1-benzyl/methyl-3,5-bis[(E)-arylidene]-piperidin-4-one and at 80°C in 1,1,3,3- tetramethylguanidine acetate [TMG][Ac] for 3–6 h and the desired products were in good yields (86–92%). When different solvents were compared, the authors employed ethanol, methanol, dioxane, acetonitrile, and toluene, as well as several ionic liquids, including [bmim] BF_4_ and [bmim]Cl. All these solvents were found to give comparatively low product yields (42–82% yield compared to 86–89 of ionic liquids in more than 5 h) and [TMG][Ac] was the best solvent, giving higher yields and shorter reaction times (92% yield in 3 h). A single regioisomer was isolated in all cases (Scheme 8).

## 1,3-Dipolar Cycloaddition in Fluorinated Solvents

2,2,2-trifluoroethanol is considered an ideal solvent and co-solvent because of its high ionizing power and strong hydrogen bond-donating ability, which provide good catalytic potential in a variety of organic transformations.

**Scheme 9:**
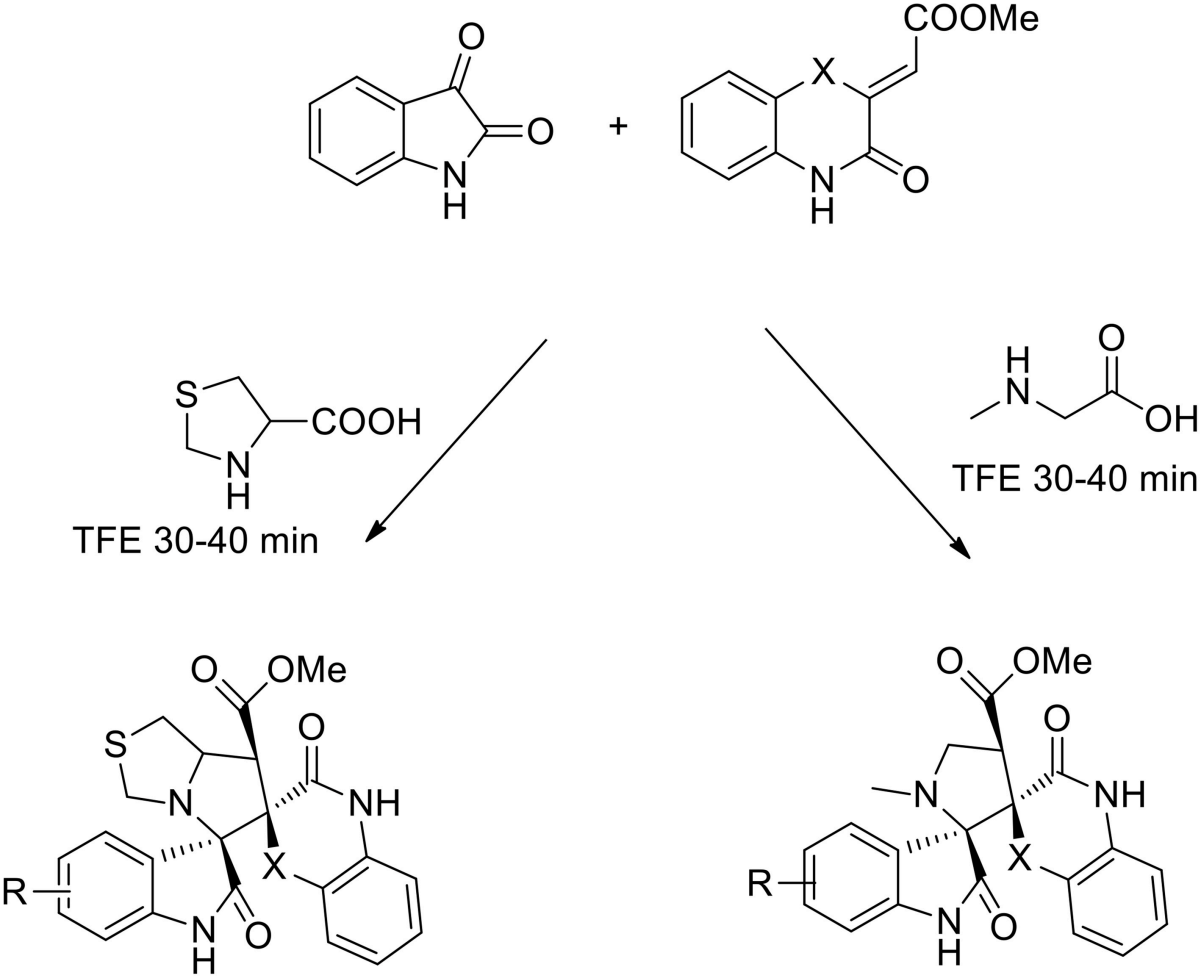
Synthesis of benzo[1,4]oxazine (X = O) and benzo[1,4]thiazine (X = S) based dispiroheterocycles *via* azomethine ylides.

The use of 2,2,2-trifluoroethanol as a recoverable greener solvent was explored by Dandia, to obtain a series of novel efficiently regio- and stereoselective dispiropyrrolidinyl/thiapyrrolizidinyl hybrid molecules via the 1,3-dipolar cycloaddition of a benzo[1,4]oxazine-derived dipolarophile, isatin and sarcosine/1,3-thiazolane-4-carboxylic acid (Dandia et al., 2015). The dipolarophile was synthesized under catalyst-free conditions from o-aminophenol and dimethyl acetylene dicarboxylate (DMAD) in good yields. The authors studied the cycloaddition reaction in different solvents (ethanol, methanol, acetonitrile, 1,4-dioxane, hexafluoro isopropanol, 2,2,2-trifluoroethanol, and toluene). The best results were obtained using 2,2,2-trifluoroethanol, which gave a single regioisomer in a higher yield and a shorter reaction time than the other solvents (92% yield). The reaction was complete in 30 min vs. the 6 h requested in presence of different solvents (Scheme 9).

**Scheme 10:**
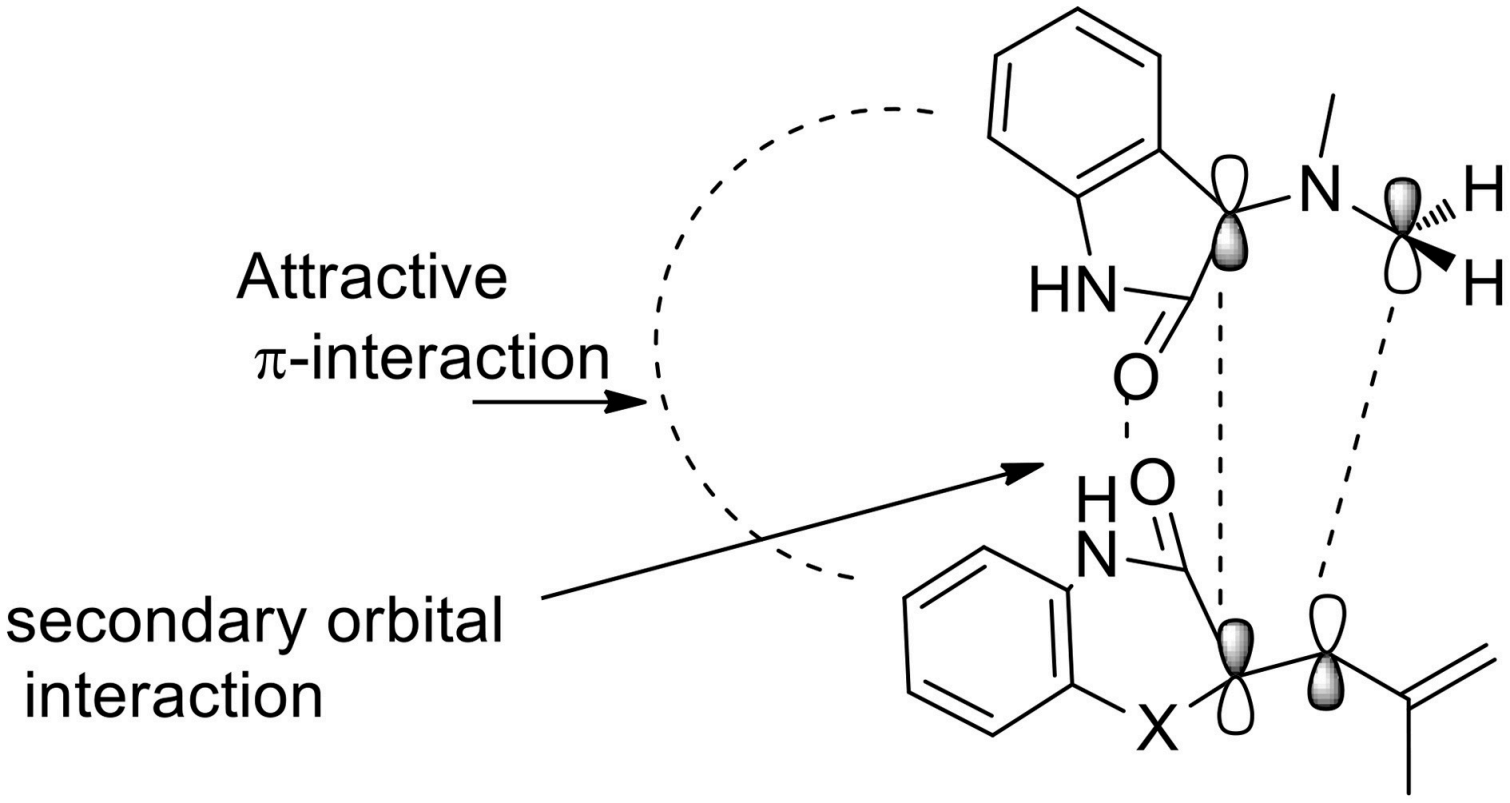
Plausible transition state.

The 1,3-dipolar cycloaddition reactions proceeded in a concerted manner, meaning that the reaction is stereospecific. In this case, the stereochemistry of alkene benzo[1,4]oxazine would influence the stereochemistry at positions 3 and 4. As described by the authors, the formation of only one diastereomer is explained by the plausible transition state (Scheme 10) which is somewhat promoted by a π-interaction between the aromatic rings as well as the secondary orbital interaction (SOI) between the ylide and the orbitals of the dipolarophile's carbonyl group.

## 1,3-Dipolar Cycloaddition in Water

The use of water in organic synthesis has stimulated several applications. Despite water's unique properties, it is still not commonly used as most organic compounds do not dissolve in water, meaning that a cosolvent is needed to increase solubility. This choice tends to diminish the advantages of low cost, no-toxicity, ease workup, and product isolation in water. Therefore, organic synthesis in aqueous media includes a large number of reactions that are performed both in homogeneous and in heterogeneous conditions (Chanda and Fokin, 2009).

As described by Sharpless et al. in their study of pericyclic cycloaddition, such reactions often benefit from working in water even when the organic reactants are insoluble in the aqueous phase. In fact, this substantial rate acceleration can be due to “on water” conditions that are created when insoluble reactants are stirred in an aqueous suspension (Narayan et al., 2005). This concept has been studied and applied to several different reactions. Even when the rate acceleration is negligible, this approach can still be considered successful as it makes product isolation easier and improves safety thanks to water's high redox stability and heat capacity.

**Figure 1:**
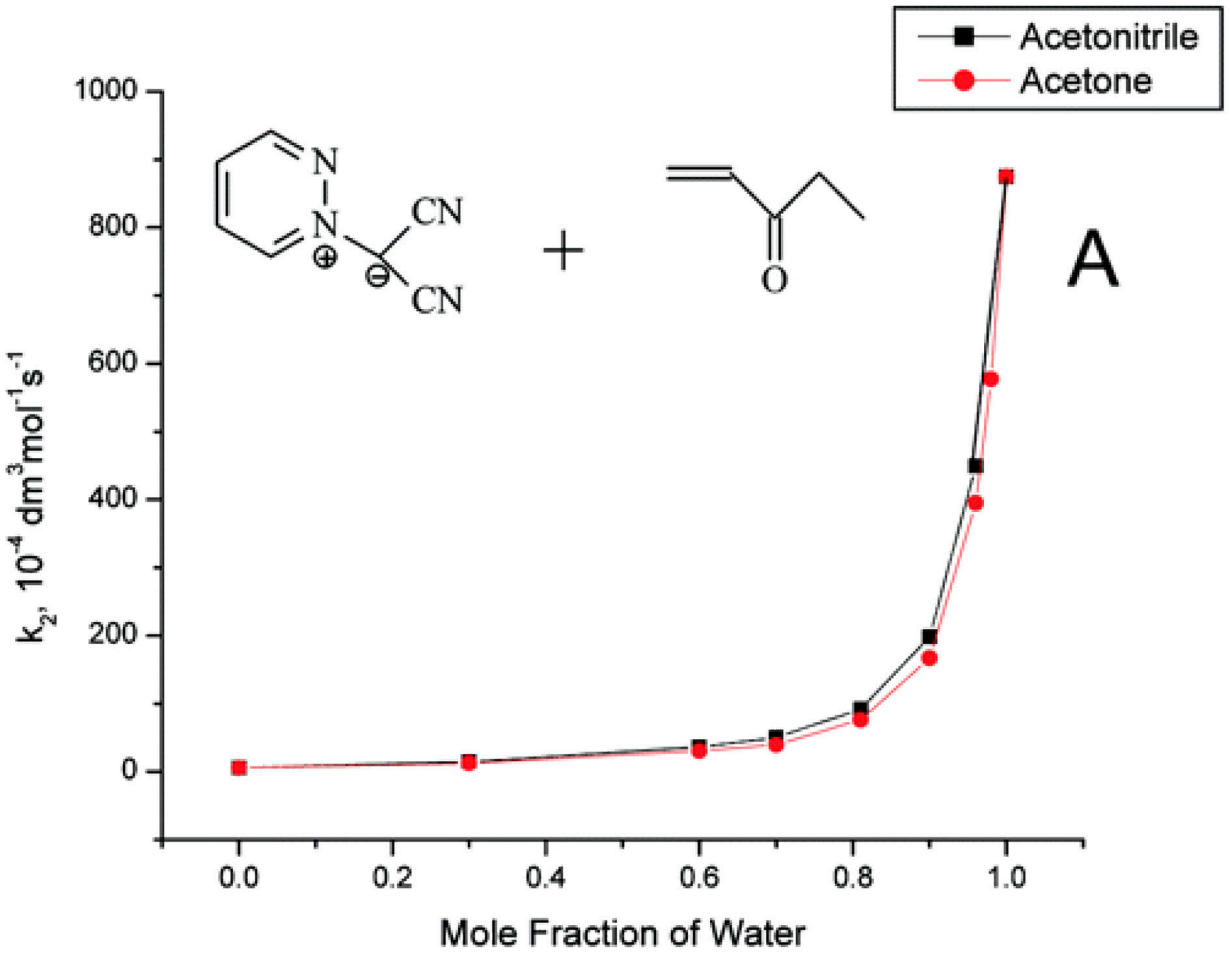
Reprinted with permission from Butler et al. (2004). Copyright (2018) American Chemical Society.

Useful information derived from comparative studies of 1,3-dipolar cycloaddition reactions in organic solvents and water. The introduction of water as a cosolvent in cycloaddition reactions in organic solvents, such as acetonitrile and acetone, gave remarkable exponential rate increases as the solvent mixture approached pure water (Butler et al., 2004). Reactions of methyl vinyl ketone, ethyl vinyl ketone, and but-3-yn-2-one with pyridazine dicyanomethanide 1,3-dipole, which is soluble in water, display rate enhancements on changing from MeCN to H_2_O (Figure 1). As observed in pericyclic cycloadditions, such as the Diels Alder reaction, the rate enhancement may be due to: (a) hydrophobic effects, which aggregate the organic reactants; (b) a lowering of the activation energy by special hydrogen bonding in the transition state; and (c) the fact that the cycloaddition transition state in water has higher polarity than its analog in organic solvents and, consequently, displays increased solvation stabilization in water (Butler et al., 2002).

**Figure 2:**
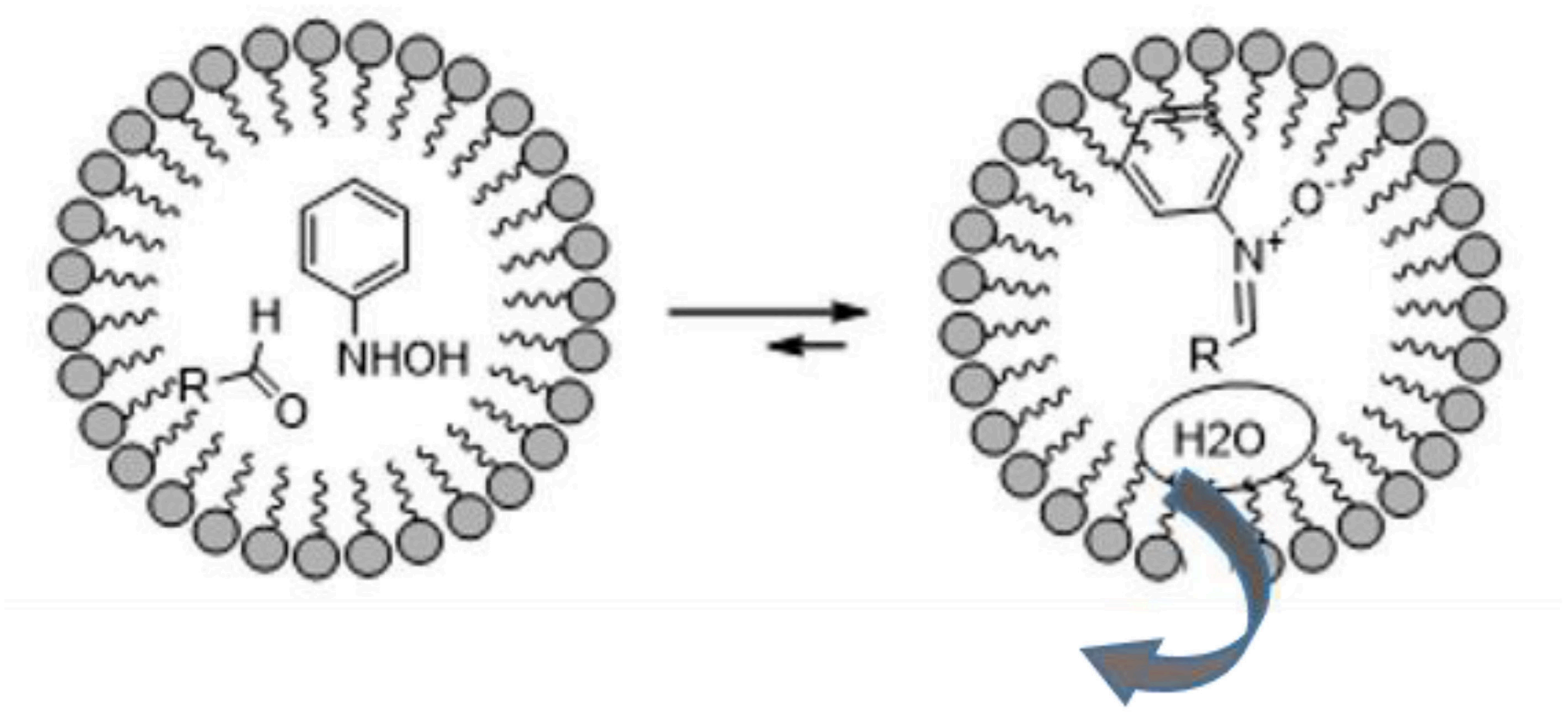
Schematic representation of nitrones synthesis in micelle.

The vast literature on the use of nitrones in the preparation of bioactive compounds pays testament to their huge synthetic potential. 1,3-dipolar nitrone-olefin cycloaddition has been used to obtain isoxazolidines, and even more complex bi- or tri-cyclic isoxazolidines, of biological interest and others that are useful synthetic intermediates for target molecules (De March et al., 1999; Fisera, 2007; Molteni, 2016). The fact that the formation of nitrones is due to the dehydration reactions of substituted hydroxylamine and carbonyl compounds drove A. Chatterjee et al. to explore the formation of nitrones in aqueous media using surfactants. As depicted in Figure 2, the micelles are hydrophobic and protect water-labile molecules from hydrolytic decomposition (Chatterjee et al., 2003).

**Scheme 11:**
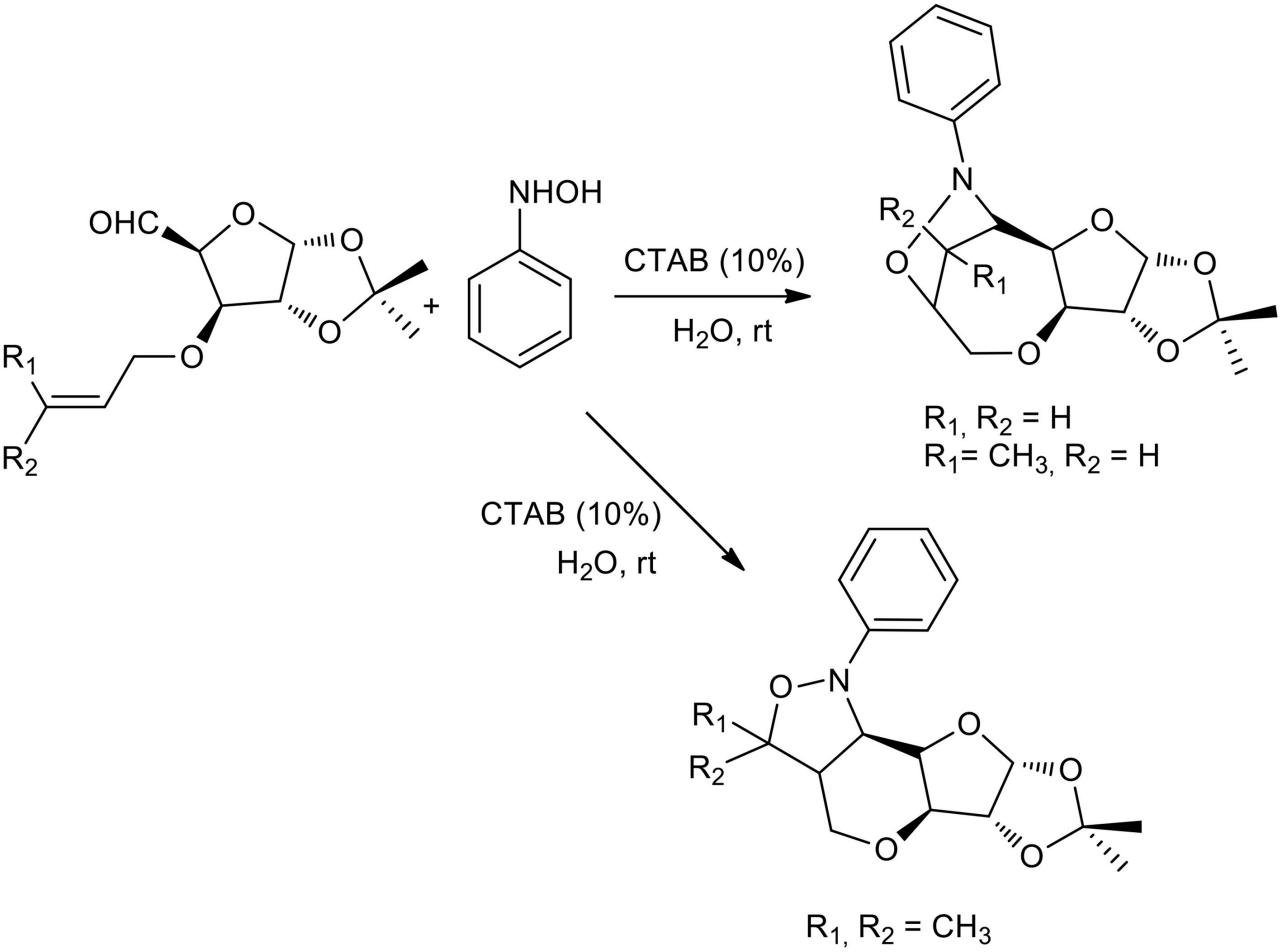
1,3-dipolar cycloaddition with furanoside-5-aldehydes derivatives and phenyl hydroxylamine.

The authors started with a model reaction between phenyl hydroxylamine and various aldehydes, in which cetyl trimethylammonium bromide (CTAB) showed better performance than sodium dodecyl sulfate (SDS) in preparing nitrones. This is presumably due to stronger binding with the substrate. Interestingly, no reaction was observed when the reaction was performed in water in the absence of a surfactant or neat. The same procedure was therefore used to obtain a stereoselective intramolecular nitrone cycloaddition (Chatterjee and Bhattacharya, 2006). Furanoside-5-aldehydes derivatives were reacted with phenyl hydroxylamine to form the corresponding nitrones in aqueous media and the reaction was catalyzed by a surfactant at room temperature. The nitrone intermediate underwent stereoselective intramolecular cycloaddition as described in Scheme 11. Interestingly, the authors observed the formation of only one of the four possible isomers in these surfactant-mediated intramolecular nitrone cycloadditions. The nitrones of 3-O-allyl glucofuranose produce bridged isoxazolidines-oxepanes, as do the nitrones of crotyl derivatives. By contrast, nitrones of prenyl derivatives produce pyrans. This may be due to methyl-methyl steric repulsion restricting the formation of the oxepane skeleton.

**Scheme 12:**
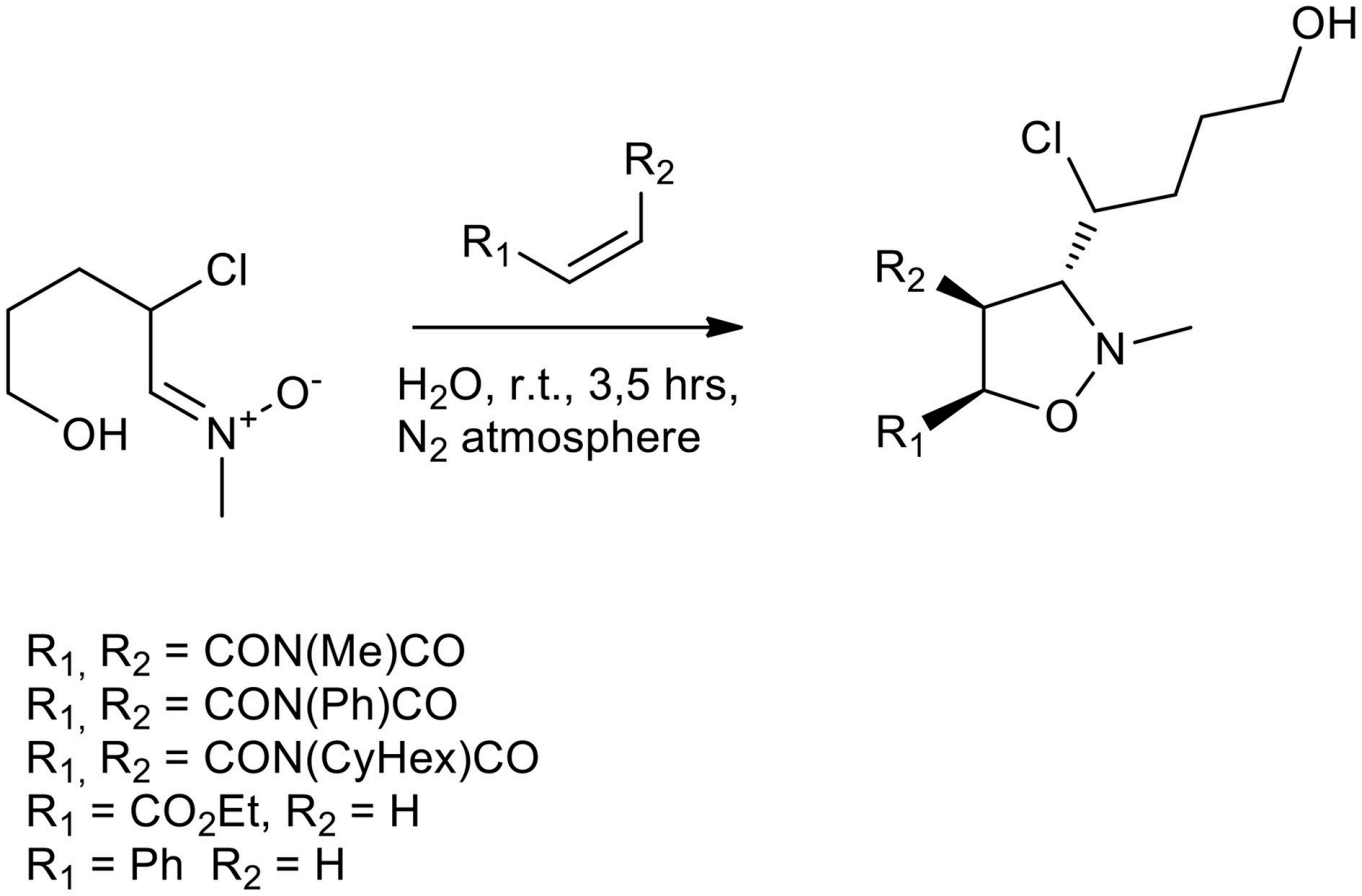
1,3-dipolar cycloaddition of *N*-methyl-α-chloronitrone.

Several other attempts to perform 1,3-dipolar cycloadditions in the presence of previously synthesized nitrones have been performed in water without the addition of a surfactant. *N*-methyl-α-chloro nitrone reacted quickly with ethyl acrylate, styrene and three different maleimides (*N*-methyl/phenyl/cyclohexyl) (Scheme 12). Yields were in the range of 91–97% and the ratio *anti/syn* was 7:3 in average (Chakraborty et al., 2010, 2012).

The 1,3-dipolar cycloaddition of nitrones in water has also been used to react two free sugar derivatives to produce pseudo-disaccharides, which bear imino-galactofuranose and galactofuranose units, in a 51% yield (Liautard et al., 2008). The reaction exploited the reactivity of water soluble nitrones with high enantioselectivity.

**Scheme 13:**
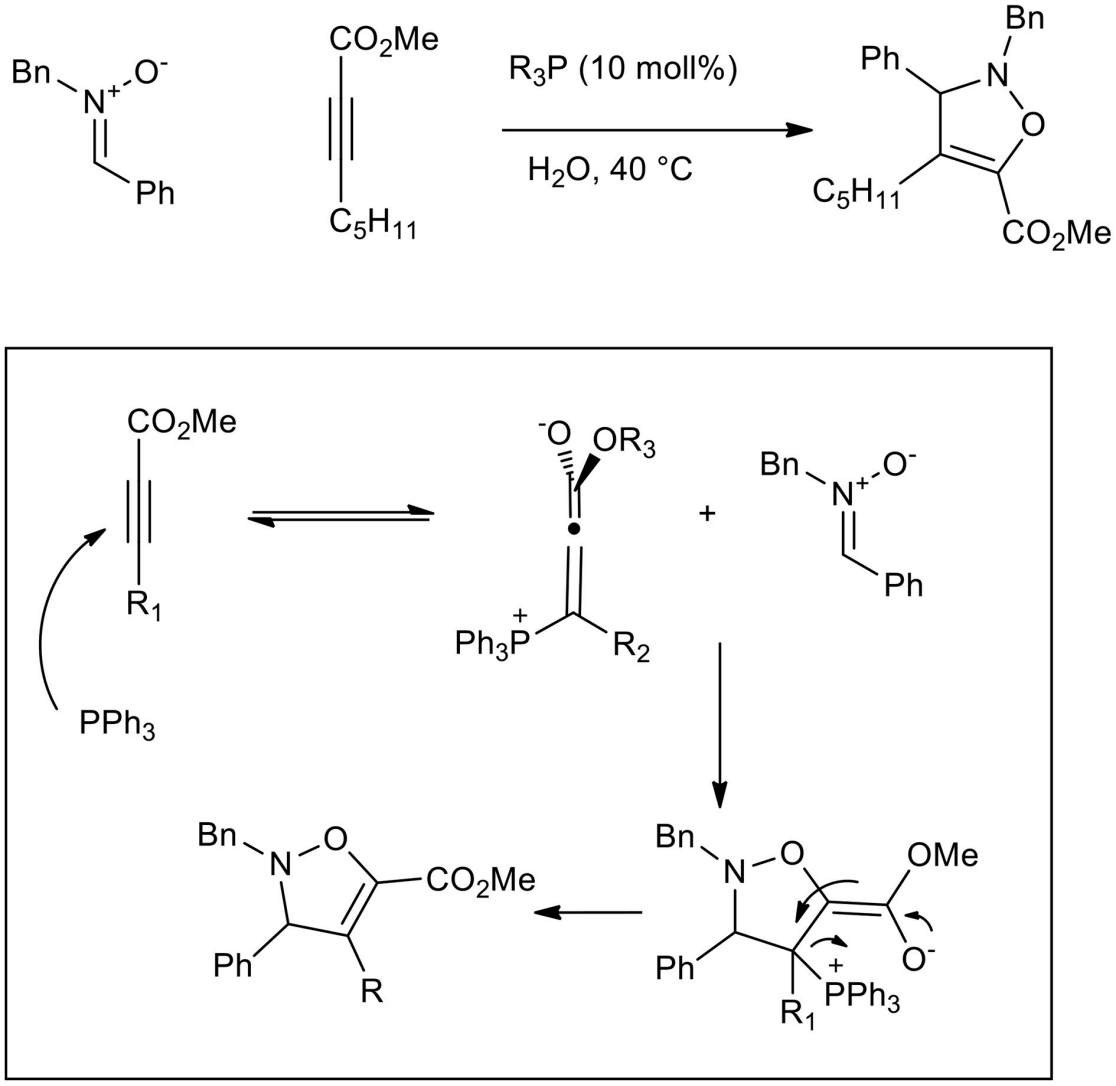
Dipolar cycloaddition of β-phosphonium (or ammonium) allenolates to obtain isoxazolidine derivatives.

Triphenylphospine and tertiary amines can be used to activate conjugated carbonyl alkynes toward 1,3-dipolar cycloaddition. In fact, β-phosphonium (or ammonium) allenolates perform as reactive dipolarophiles in aqueous 1,3-dipolar cycloadditions and have been used to give 2,3-dihydroisoxazole (Scheme 13) (Gonzalez-Cruz et al., 2006). The reaction increased the reaction yield up to 68% when LiCl was used and interestingly only one regioisomer was isolated, while no reaction was observed in toluene and DCM. By comparison with tertiary amines, PPh_3_ showed the best catalytic activity in the presence of aromatic nitrone (68% yield vs. 59%), while quinuclidine was more active with aliphatic nitrones. As has already been reported, reagents solubility in water is not required as they react in suspension (“on water”).

**Scheme 14:**
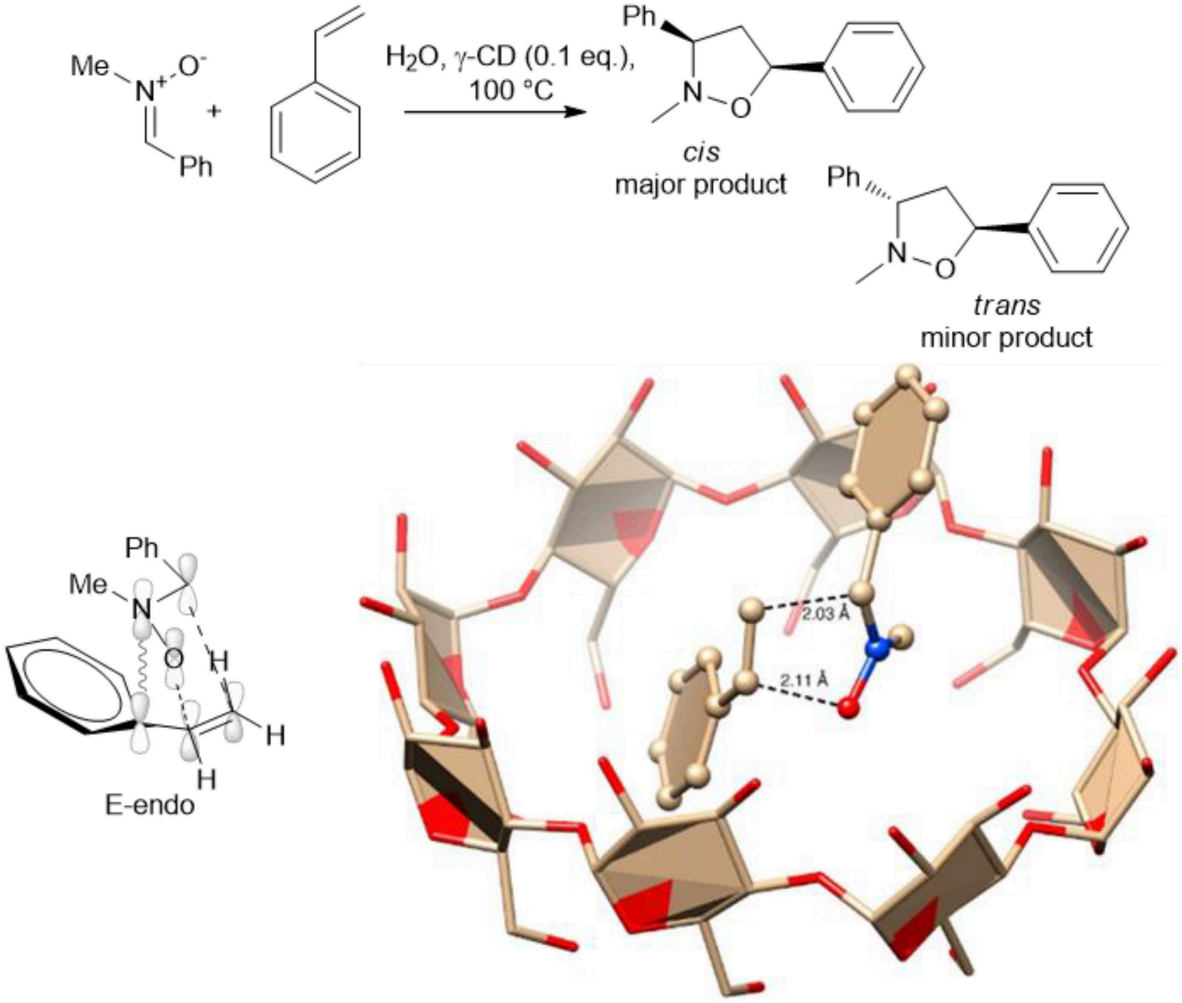
Schematic representation of synthesis of 3,5-diarylisoxazolidines via the 1,3-dipolar cycloaddition in presence of γ-CD and plausible transition stage to obtain the major E-*endo* derivative. The image was reprinted adapted with permission from Floresta et al. (2017). Copyright (2018) American Chemical Society.

A catalytic amount of γ-cyclodextrin (γ-CD) has been used by G. Floresta et al., to produce 3,5-diarylisoxazolidines via the 1,3-dipolar cycloaddition of several *p*-substituted nitrones, styrenes and cinnamate derivatives (Floresta et al., 2017). The cyclodextrin cavity acted as a reactor and heating at 100°C for 8–12 h gave a set of derivatives in good yields with moderate to excellent diastereoselectivity. Interestingly, β-CD was inactive as a nano-reactor, while γ-CD was found to efficiently form inclusion complexes. The formation of the complex was confirmed by HR FT-ICR MALDI-MS Studies and NMR spectra. The authors rationalized by an *in silico* study the cycloaddition process and an E-*endo* transition state and that the cis major adduct is the more reactive rotamer of the nitrone. The calculation suggests that the transition stage is positioned within the CD reactor, as depicted in Scheme 14.

**Scheme 15:**
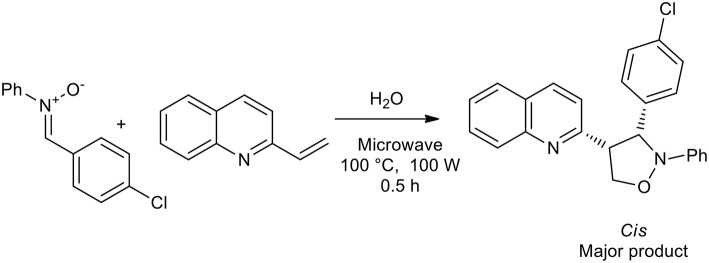
Synthesis of 5-substituted isoxazolidines with nitrones in water under microwave irradiation.

In 2008, an interesting publication demonstrated the influence that the solvent can have on the production of isoxazolidine. Alkenylazaarenes were utilized as dipolarophiles for the preparation of 4-substituted and 5-substituted isoxazolidines with nitrones giving high regioselectivity under a range of reaction conditions. Reaction in water under microwave in a sealed vessel gave in high to excellent yields only 5-substituted isoxazolidines, while the 4-substituted analogs were obtained as the major products when a Lewis acid, TMSOTf, was used as the catalyst in DCM at room temperature (Scheme 15) (Tong et al., 2018).

Oxindole derivatives that bear a spirocyclic quaternary stereocentre at the C3 position are interesting heterocyclic motifs generally found in a number of natural products and drugs.

**Scheme 16:**
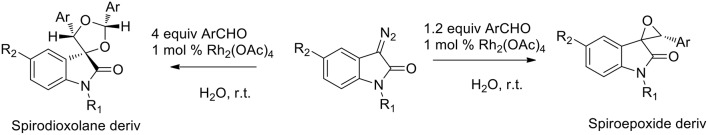
Schematic representation of spiro-indole synthesis from 3-diazooxindole derivatives.

Several green approaches for the synthesis of spiro-indole have been described as occurring in the presence of water as a solvent. Despite the common use of halogenated organic solvents, the “on-water” generation of carbonyl ylides using carbenoids was described in 2015 (Muthusamy and Ramkumar, 2015). The proposed synthesis catalyzed by rhodium(II) acetate dimer, afforded spiro-oxiranes from carbonyl ylide dipoles and diazoamides (3-diazooxindoles) (Scheme 16). Spiro-dioxolanes were obtained with complete diastereoselectivity when carbonyl ylides were obtained using aromatic aldehydes with electron-withdrawing substituents, while with electron-donating substituents the 1,3-dipolar cycloaddition reactions were observed.

**Scheme 17:**
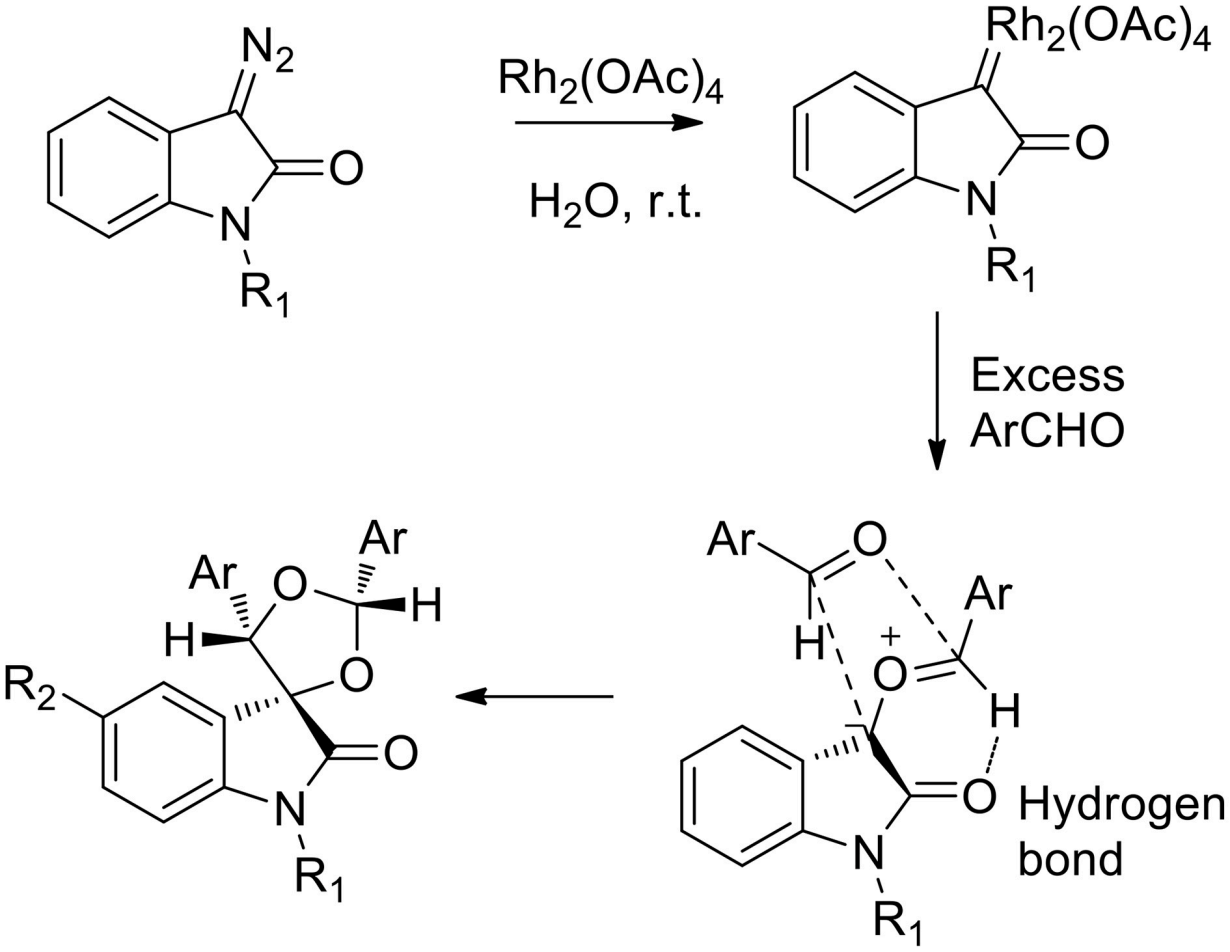
Proposed mechanism.

The reaction afforded *cis* products in both the spiro-oxirane and spiro-dioxolane syntheses. In the presence of 1.2 equivalents of aromatic aldehyde, the acyclic carbonyl ylide underwent the intermolecular stereoselective dipolar cyclization in a particular conformation, which involved an intramolecular hydrogen bond, to give epoxides (Scheme 17). When an excess of aldehyde was used (4 equivalents), the reaction happened “on water” as the nucleophilic addition of water was not observed to occur. Nevertheless, the free interfacial-water OH groups may stabilize the possible transition state.

A three-component, 1,3-dipolar cycloaddition of *in situ* generated azomethine ylides from isatin derivatives and benzylamine, with benzylideneacetone can be employed to give spiro-pyrrolidine-oxindoles (Peng et al., 2014). This reaction can be performed in water to give the desired product in a 23% yield and a 68:32 regioisomeric ratio.

**Scheme 18:**
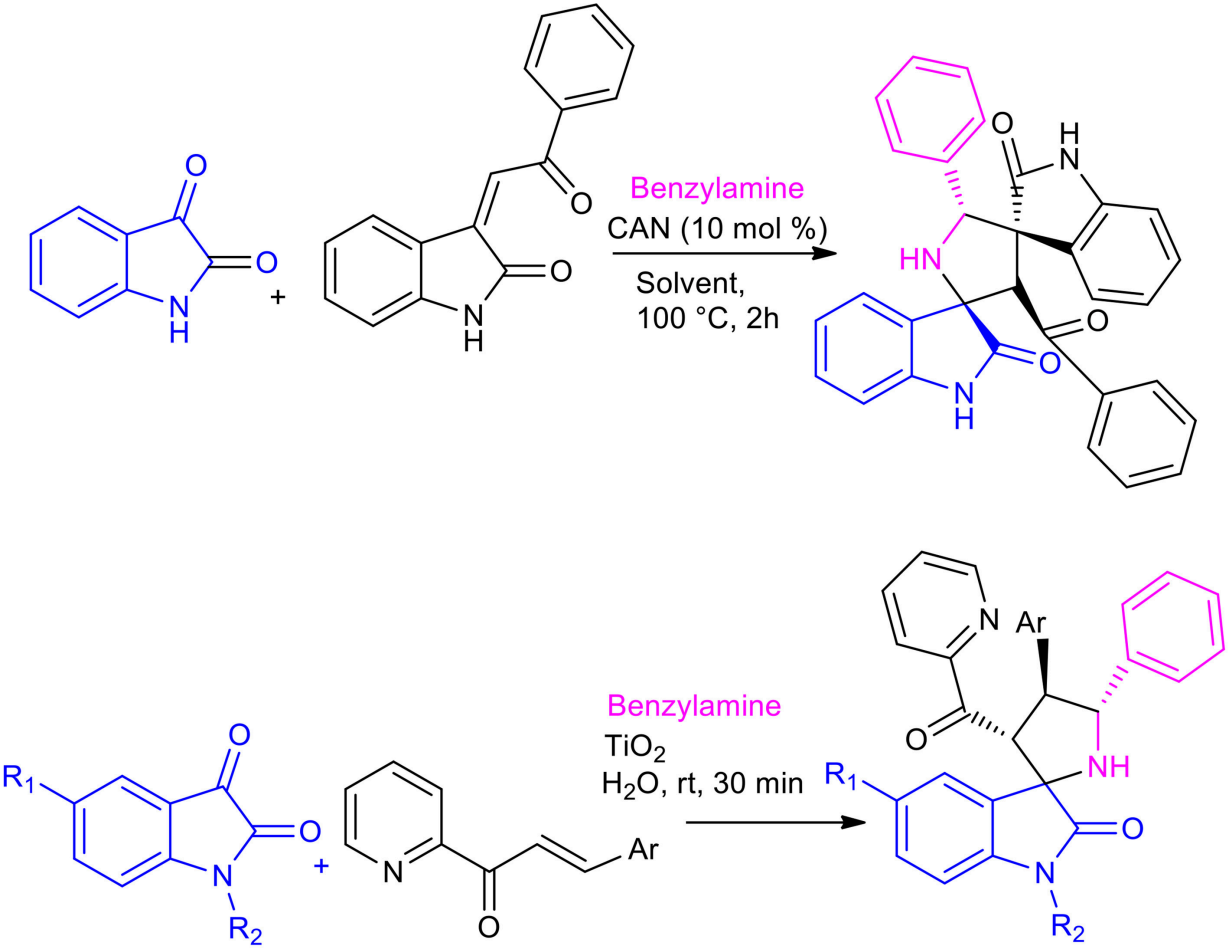
Synthesis of spiroindole by 1,3-dipolar cycloaddition with TiO_2_ in water and with CAN in solvent.

The use of Lewis acids, such as Ceric Ammonium Nitrate (CAN) or TiO_2_, in the formation of spirooxindoles in water has been investigated, giving excellent results (Scheme 18) (Ramesh et al., 2016, 2018). The 1,3-dipolar cycloaddition was performed with 1 mol% of CAN in 2 h, while the reaction was complete after 30 min when heterogeneous TiO_2_ nanoparticles were used. Both reactions showed excellent regioselectivity and stereoselectivity, and the TiO_2_ nanocatalyst was reused 5 times without losing catalytic activity. 20 different spirooxindole-pyrrolidines were obtained, in 65–90% yields with CAN and the reaction showed increased average yield in the presence of TiO_2_.

**Scheme 19:**
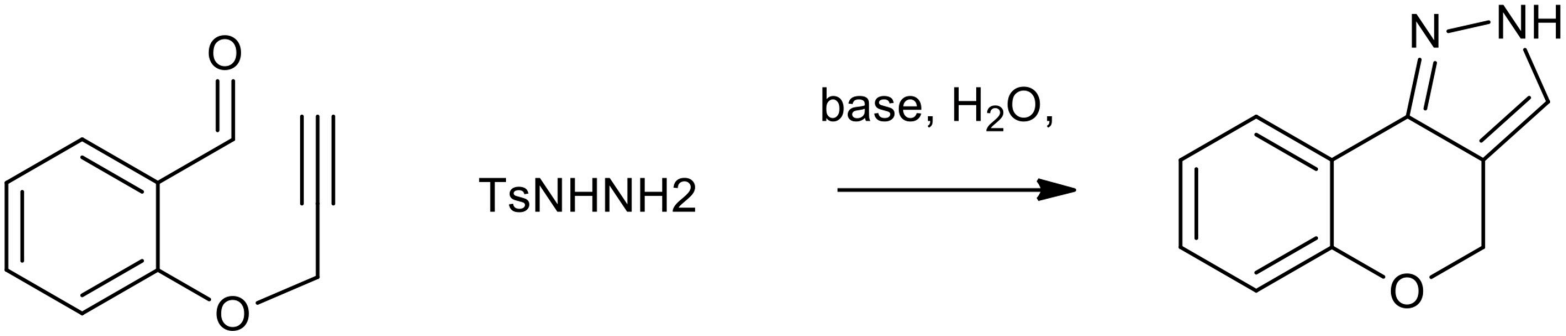
Synthesis of benzopyranopyrazole from propargylated salicylaldehydes and tosyl hydrazine.

Diazoalkanes can be generated from the decomposition of tosylhydrazones and are efficient 1,3-dipoles exploitable for the synthesis of several nitrogen containing heterocycles (Munro and Sharp, 1984). An intramolecular 1,3-dipolar cycloaddition strategy for rapid access to pyrazoles or triazoles makes use of the *in situ* generated diazomethanes, in a two-step sequence, in which diazomethanes undergo smooth cycloaddition with alkyne or nitrile moieties (Padwa and Ku, 1980). As described in Scheme 19, this process can be used as a step-economical route to benzopyranopyrazole from propargylated salicylaldehydes and tosyl hydrazone. This reaction, which is usually performed in DMF, can be performed in water with K_2_CO_3_, providing excellent yields (more than 80%).

## Organo-Catalyzed and Catalyst-Free 1,3-Dipolar-Cycloaddition

A novel organocatalytic asymmetric 1,3-dipolar addition has been proposed by MacMillan, that explored the synthesis of oxazolidine starting from nitrones and α,β- unsaturated aldehydes (Jen et al., 2000). In the presence of different chiral imidazolidinone**·**HCl the reaction between crotonaldehyde and *N*-benzyl phenyl nitrone showed moderate to high yield (45–77%) and *ee* (42–93%); when a Brønsted acid was added as co-catalyst, the efficient iminium activation could increase yield (98%) and *ee* (94%).

**Scheme 20:**
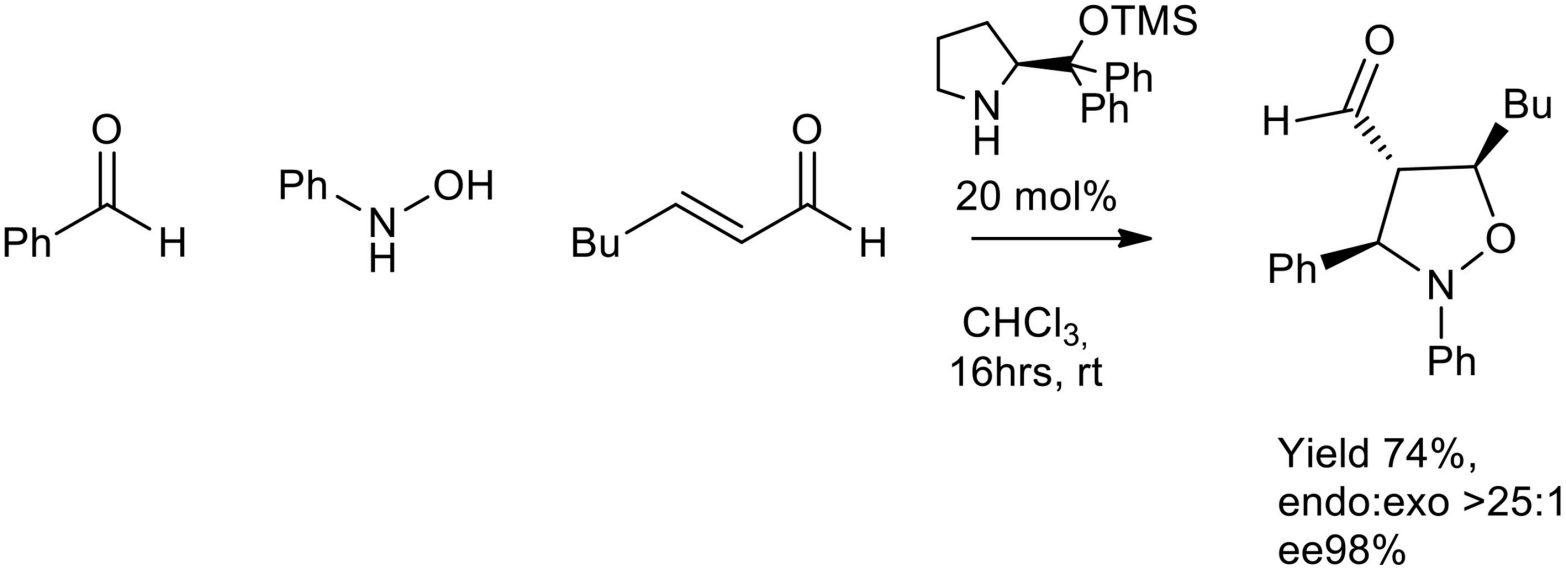
Enantioselective synthesis of substituted isoxazolidine.

In 2007 Córdova et al. proposed an enantioselective organocatalyzed synthesis of substituted oxazolidine. *N*-arylhydroxylamines, aldehydes, and α,β-unsaturated aldehydes were reacted in the presence of a chiral organic catalyst. The *in-situ* generated nitrone reacted with activated enal, in chloroform or THF at room temperature (16 h) to give the isoxazolidine as a single diastereomer (>25:1 *endo:exo*). TMS protected diarylprolinol gave *ee* % up to 98% (Scheme 20) (Rios et al., 2007).

**Scheme 21:**
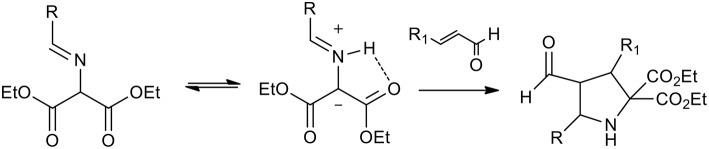
Schematic representation of iminoesters as the precursors of stabilized azomethine ylides.

Organo-catalyzed (3+2) dipolar cycloadditions of azomethineylide with various dipolarophiles has been the object of intense investigation. Several publications studied iminoesters as the precursors of stabilized azomethine ylides, because, as described in the scheme, they can undergo thermal tautomerism to produce azomethine ylides (Scheme 21).

**Scheme 22:**
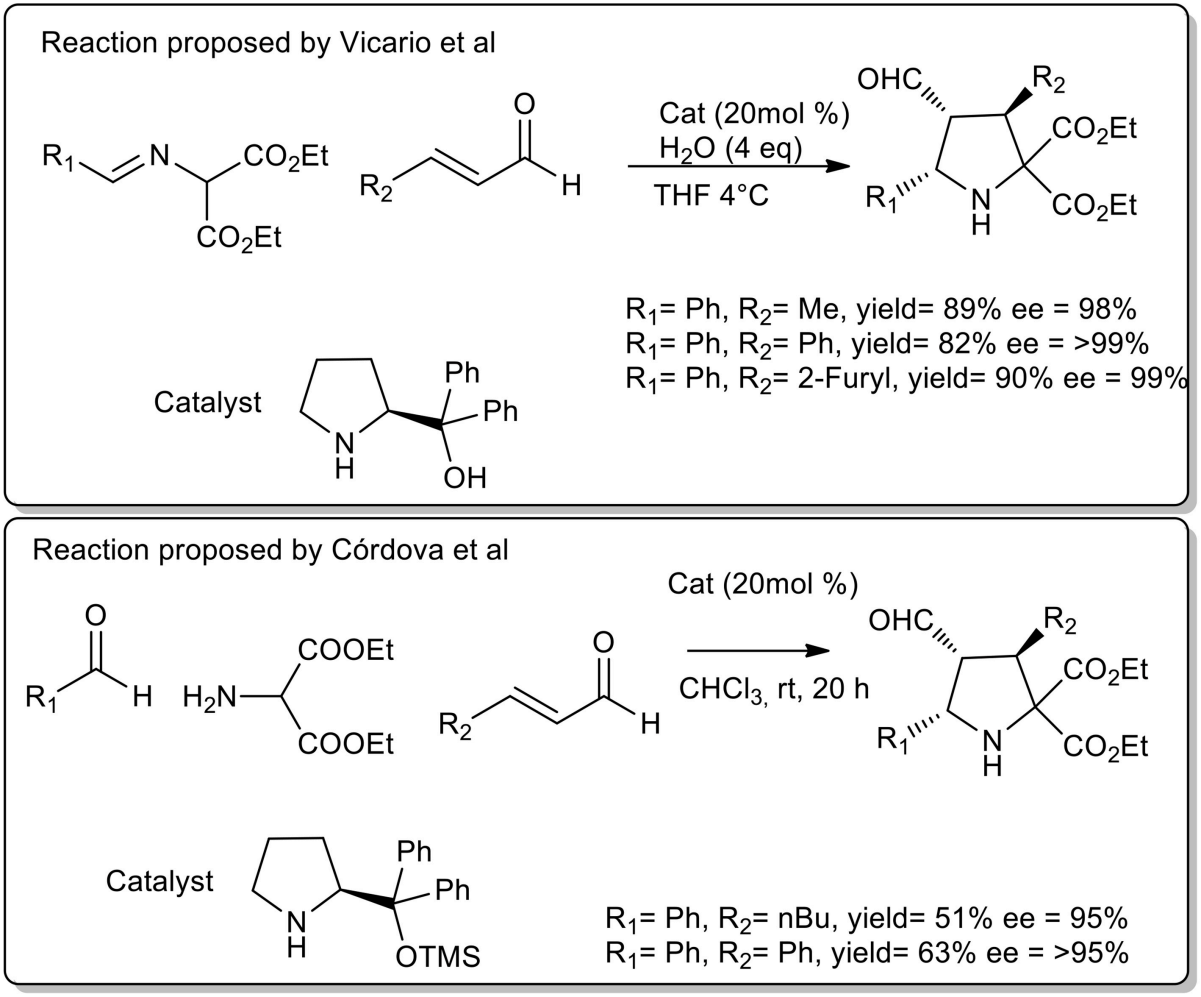
Enantioselective synthesis of substituted pyrrolidines.

Vicario et al. observed that cycloaddition of imine and crotonaldehyde catalyzed diphenylprolinol provided a single *endo* isomer with excellent enantioselectivity (Vicario et al., 2007). The authors highlighted the fundamental role of free OH groups on the catalyst and the acceleration rate by water addition. The optimized protocol was performed at 4°C in THF. Similarly, Cordoba et al. proposed the synthesis of pyrrolidine in CHCl_3_, at room temperature with protected prolinol. As described in Scheme 22, a slightly lower yield and selectivity were observed (Ibrahem et al., 2007).

**Scheme 23:**
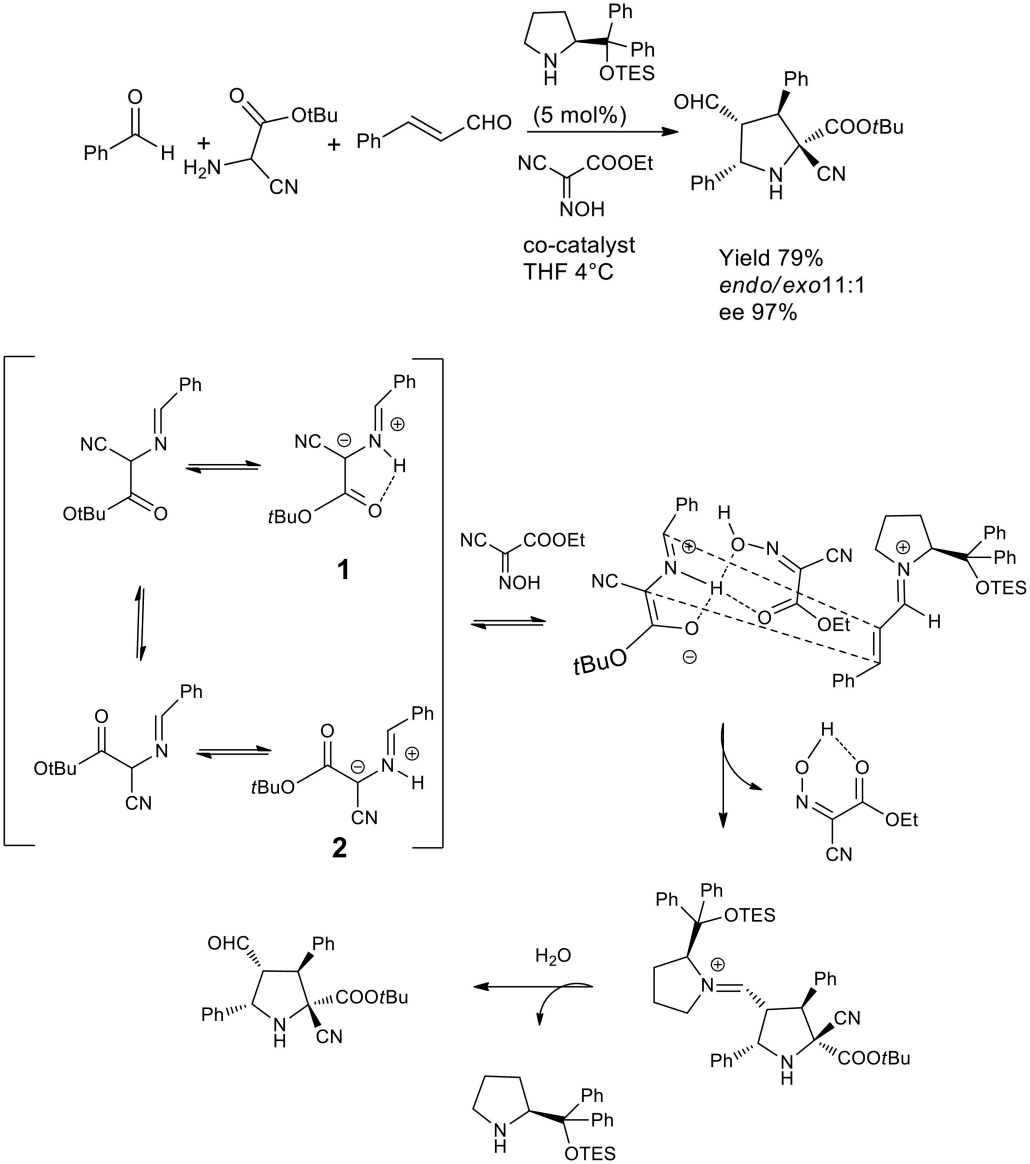
One pot Three component catalytic asymmetric synthesis of pyrazolidine.

An elegant example of 1,3-dipolar cycloaddition in asymmetric catalysis has been published by the Córdova group, which proved that a hydrogen bond donating network with a co-catalyst was to direct the cycloaddition by locking the conformation of the intermediate so to achieve a highly selective reaction (Lin et al., 2011). This dynamic one pot reaction was directed to cycloaddition in THF or DMF and the presence of hydrogen bond donating molecules such as oximes was favorable for the acceleration of the reaction when the substrate was cyanoacetate or α-cyanoglycine. Compared to the previously described approach, this one pot reaction generated four contiguous chiral centers including a quaternary carbon. As described in the scheme, the proposed mechanism involves the prototropy of the imine cyanoglycine so that the H bond activate the iminium salt and lock is conformation as **1** (see Scheme 23) stereoselective ccloaddition from the Re face of intermediate **3** by and *endo* mechanism.

**Scheme 24:**
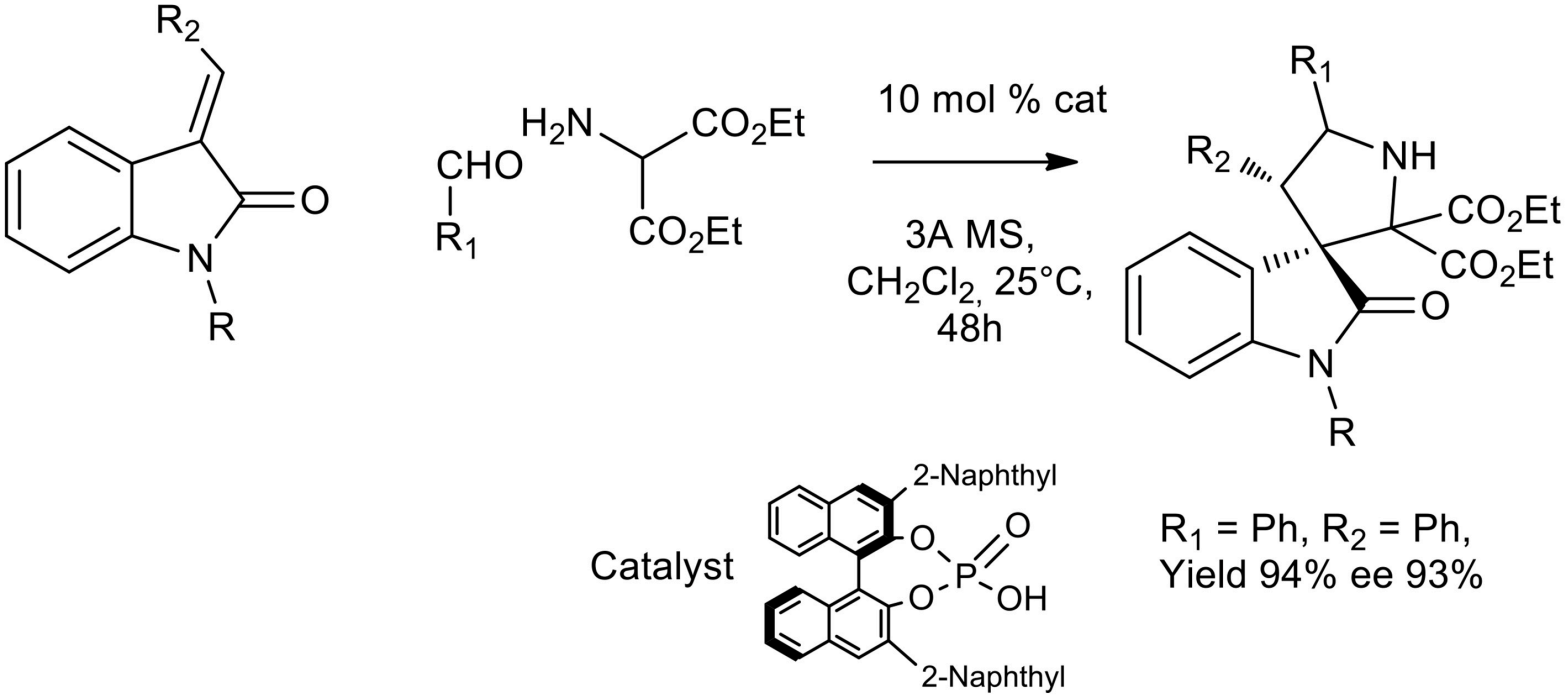
Organocatalytic synthesis of spiro[pyrrolidin-3,3′-oxindoles].

Another recent approach to activate different dipolarophiles other than unsaturated aldehydes was pursued by Chen et al. and chiral phosphoric acids were successfully exploited to obtain pyrrolidine or spirooxindole (Chen et al., 2009; Chang et al., 2016). Excellent yield and enantioselectivity were obtained in DCM at room temperature with chiral sterically hindered phosphoric acids for the imine activation (see Scheme 24).

**Scheme 25:**
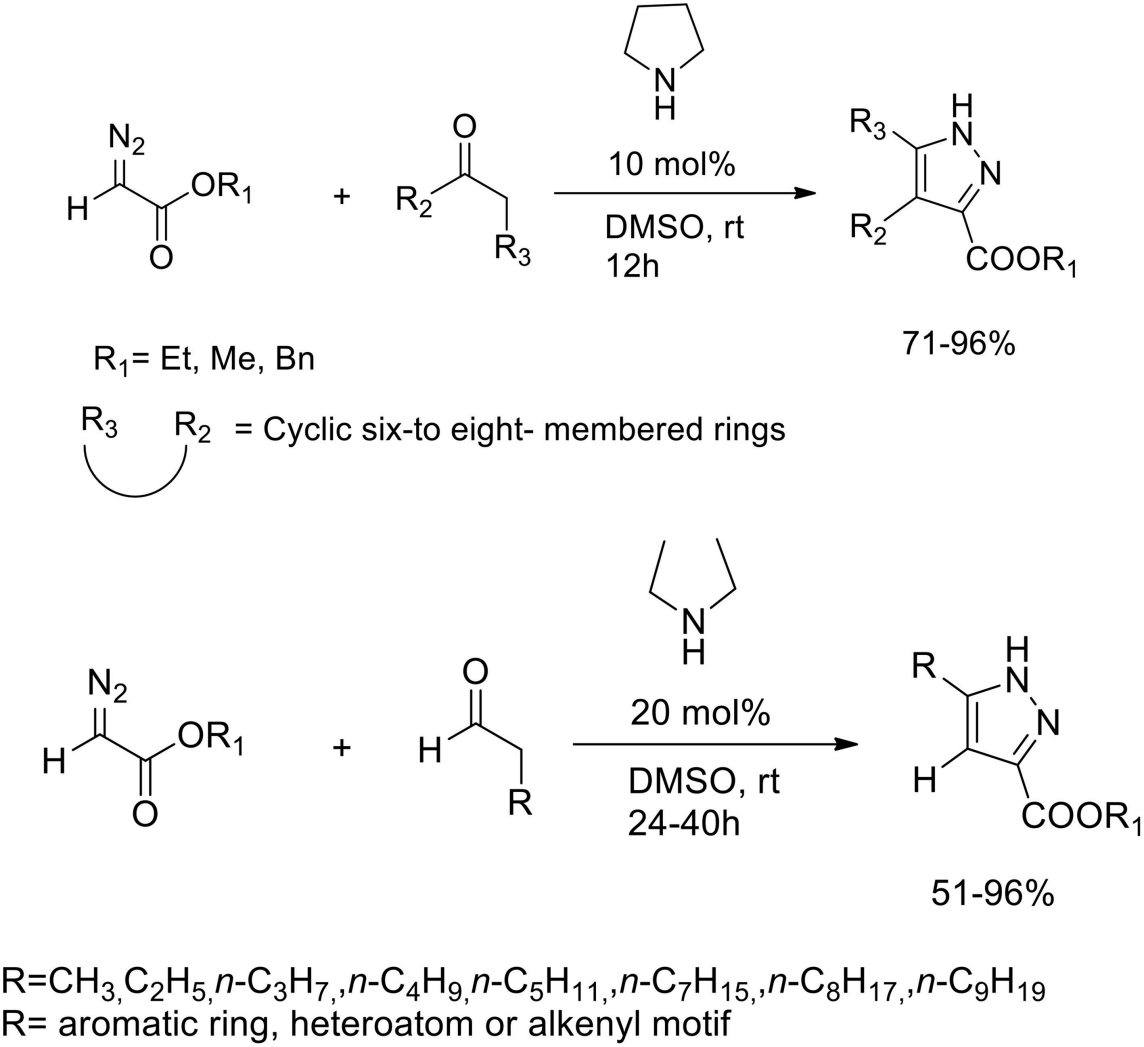
Polysubstituted pyrazoles synthetic route from diazoacetates and carbonyl compounds.

Pyrazoles are known to be potent insecticides and herbicides, and have been also studied for their anti-tumor, anti-inflammatory, anti-microbial and anti-psychotic properties. Diazo compounds may react with alkynes to provide efficient synthesis of pyrazole via 1,3-dipolar cycloaddition. The 1,3-dipolar cycloaddition of alkynes to electron-rich diazo compounds has been described, whereas the intermolecular 1,3-dipolar cycloaddition of alkynes with electron-poor diazocarbonyl compounds is much less often reported because of the to the high HOMO–LUMO energy difference between alkynes and diazocarbonyl compounds. In presence of Lewis acid or transition metals LUMO of the alkyne dipolarophiles is lowered. Wang et al. (2013) developed an organocatalytic inverse-electron-demand [4+2] cycloaddition between diazoacetates and various carbonyl compounds. Secondary amines we employed as “green promoters,” to catalyze the cycloaddition reaction and produce the target pyrazole ring (Scheme 25). Pyrrolidine was selected as the most effective catalyst and DMSO as best solvent for this transformation. The optimized protocol was performed in DMSO at room temperature with 10 mol% of pirrolidine and a 1:2 ratio of diazoacetates and carbonyl compounds.

When the reaction was performed with unsymmetrical cyclic ketones, high levels of regioselectivity were achieved. Nonetheless, the authors discovered that in the reaction between diazoacetates and aldehydes, it performed better in presence of acyclic secondary diethyl amine catalysis. Moderate-to-excellent yields of the corresponding adducts were obtained also varying the side-chain of the carbonyl group.

**Scheme 26:**
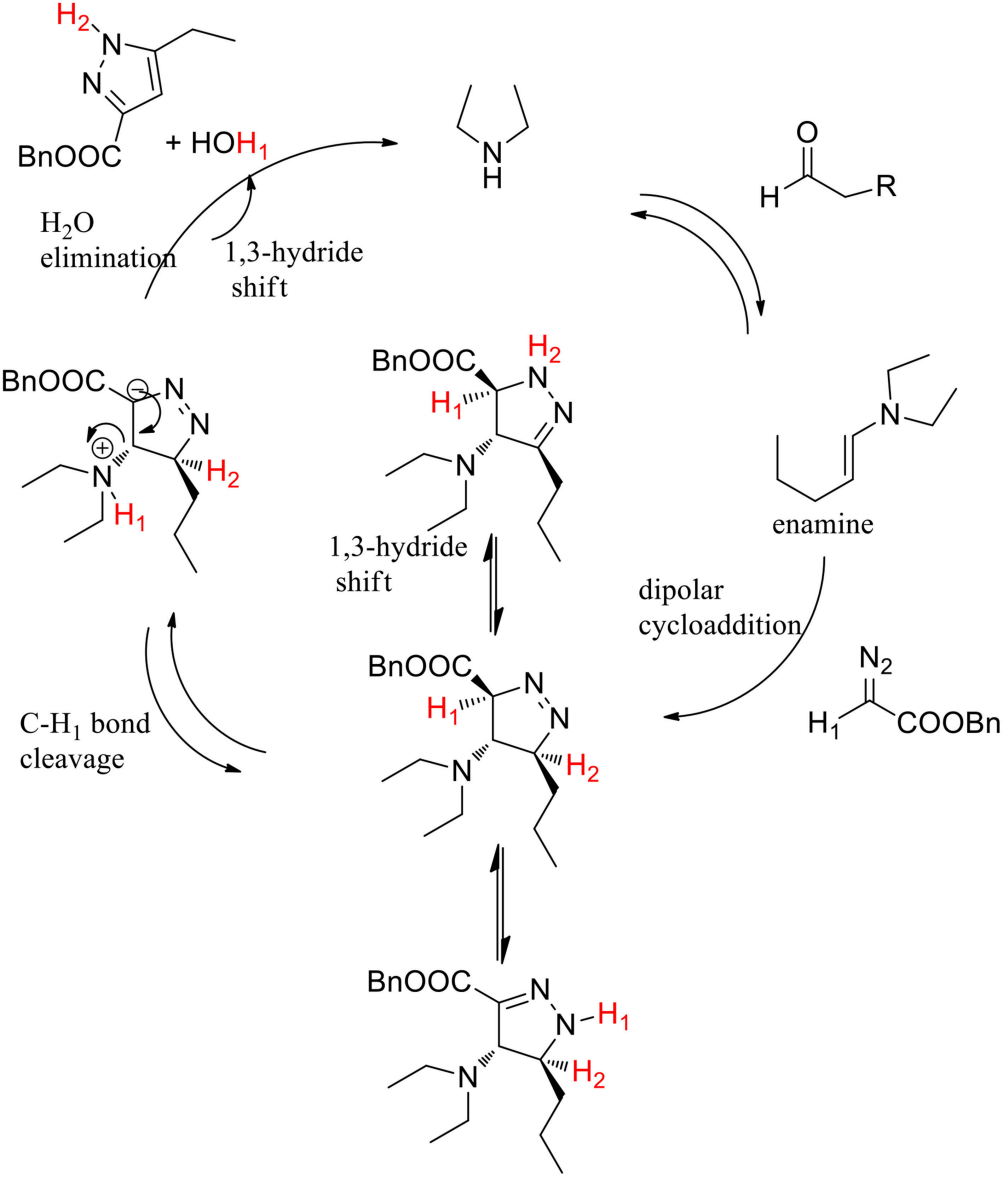
Postulated reaction pathway.

The hypothesis of selective C-H_1_ bond-breaking was confirmed by a deuterium labeling experiment. The enamine-promoted cycloaddition reaction with α-deuterated benzyl diazoacetate yielded the final compound without any deuterium being incorporated into the pyrazole ring, which supports the hypothesis of a selective C-H_1_ bond breaking. The authors supposed that the C-H_1_ bond is activated by the adjacent electron-withdrawing ester group, toward the selective cleavage over the C-H_2_ bond. The final product derived from an elimination step followed by tautomerization (Scheme 26).

**Scheme 27:**
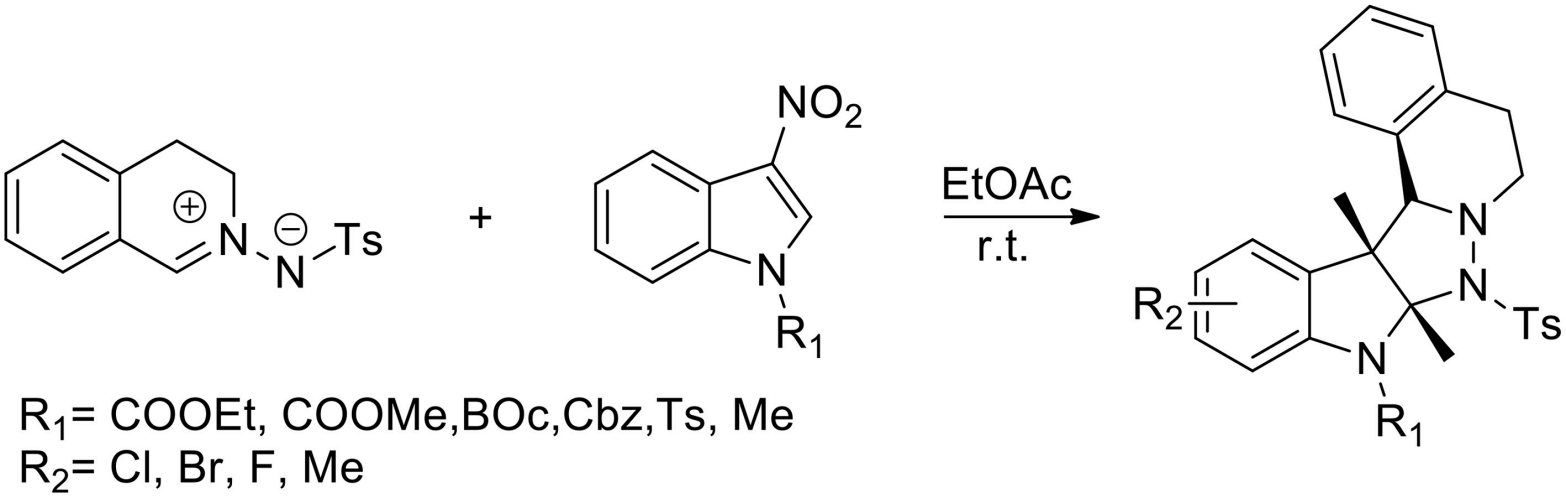
Synthesis of polycyclic tetrahydroisoquinoline derivatives.

Liu et al. (2017) have presented the first catalyst-free 1,3-dipolar cycloaddition of C,N-cyclic azomethine imines and 3-nitroindoles to prepare highly functionalised, five-ring-fused tetrahydroisoquinolines, which feature an indoline scaffold with excellent diastereoselectivity. The reaction performed the use of catalysts or additives and more than 95% yield was obtained in EtOAc (Scheme 27). In the presence of 20 mol% Cu(OTf)_2_ in CHCl_3_ the cycloaddition reaction afforded the corresponding product with only 23% conversion after 24 h at room temperature. When Ni(OAc)_2_·4H_2_O was used in CHCl_3_, the conversion was enhanced to 89%. *N*-tosyl and *N*-alkoxycarbonylated protected, 3-nitroindoles performed very well giving in high yields the corresponding cycloadducts. Because of its reduced electrophilicity *N*-Methyl-protected 3-nitroindole failed to undergo the transformation. The versatility of this method was demonstrated with a list of structurally different C,N-cyclic azomethine imines.

1,2,3-triazoles have a wide range of applications as potential bioactive compounds and are often used in drugs synthesis. A general and efficient method for their fabrication came in the early 2000s with the novel concept of “click” chemistry and the Cu-catalyzed alkyne–azide cycloaddition reaction provides the regioselective formation of 1,4-disubstituted 1,2,3-triazoles. The metallo-catalyzed alkyne–azide cycloaddition reaction for the formation of 1,5-disubsituted 1,2,3-triazoles was published later. However, the reactions mentioned above have made use of heavy metals, which has limited their practical applications.

**Figure 3:**
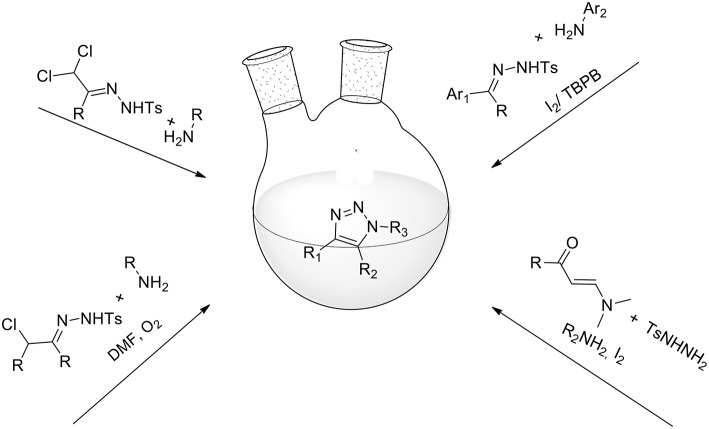
“Metal-free” and “azide-free” syntheses of triazoles.

Alternative synthetic pathways for 1,2,3-triazoles have also been developed and 1,2,3 triazoles have freely been obtained from a combination of azides with a range of reaction partners: e.g., cycloadditions of either β-keto esters or nitriles to azides catalyzed by secondary amines (Costa et al., 2017), cycloaddition of a triple domino sequence of reactions between azide, amine, and 5-bromo-2-furylcarbinol (Yang et al., 2015), the reaction of enols and enamines with azides (Blastik et al., 2018), nitro methylene-based three-component synthesis (Thomas et al., 2014), and others. However, these methods entail the use of organic azides or sodium azides, which are difficult to handle and toxic, particularly on a large scale. In a 2017 review, Ahmed (Ahmed et al., 2017) presented some less well-known synthetic protocols for 1,2,3-triazoles under azide-free and metal-free environments (Figure 3).

## Light Induced

**Scheme 28:**
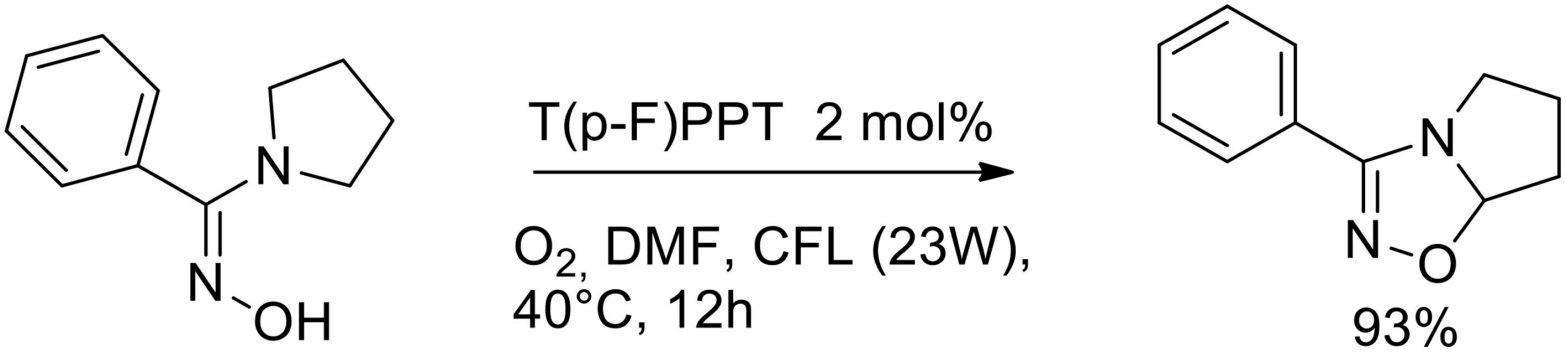
Synthesis of bicyclic 1,2,4-oxadiazolines.

The importance of aza-heterocycles relates to their presence as natural products, drugs and biologically relevant compounds. A variety of methods have been developed for the synthesis of 1,2,4-oxadiazolines, and these are typically carried out via the [4+2] cycloaddition of an imine with a nitrile oxide (generated *in situ* from hydroxamoyl chloride or nitroalkane). The design of a new synthetic path of 1,2,4-oxadiazolines, especially greener methods, is highly desirable because many of these methods suffer from one or more drawbacks. Soni et al. (2018) have very recently presented a greener method for the synthesis of 1,2,4-oxadiazolines via an intramolecular oxidative cyclisations of amidoximes in the presence of an organocatalyst and molecular oxygen. The authors optimized the reaction conditions to give 3-phenyl- 5,6,7,7a-tetrahydropyrrolo[1,2-d][1,2,4]oxadiazole from phenyl(pyrrolidin- 1-yl)methanone oxime, which was used as a model substrate. The optimized conditions involved 2 mol% of an organophotocatalyst, 2,4,6- tris(4-fluorophenyl)pyrylium tetrafluoroborate [T(p-F)PPT] at a 0.2 M concentration in DMF under an atmosphere of molecular oxygen. Visible-light irradiation was provided by a compact fluorescent lamp (CFL, 23 W). Organophotocatalyst can reduce the drawbacks of transition metals related to toxicity and the low residues admitted in pharmaceutical products (Scheme 28).

**Scheme 29:**
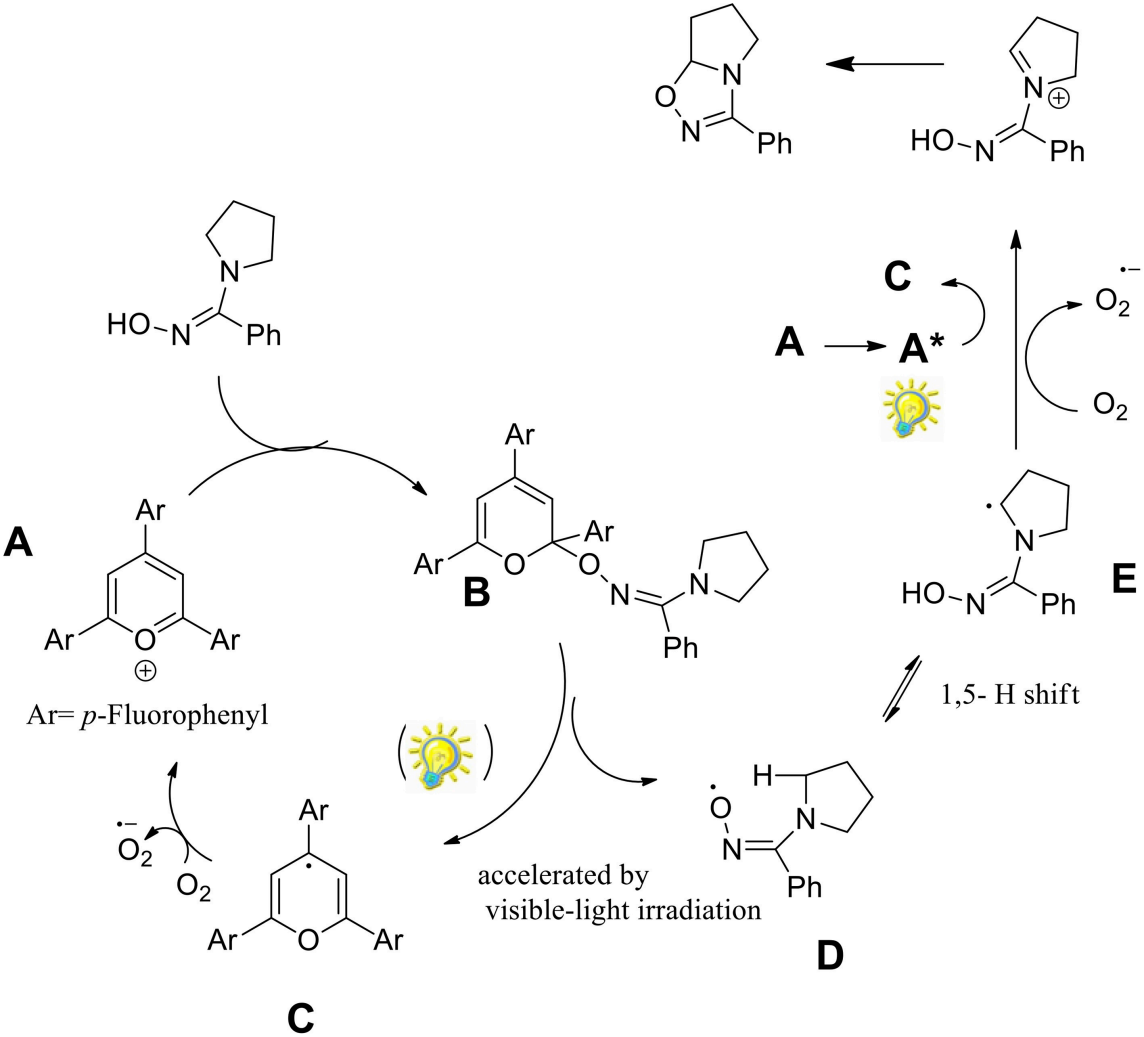
Plausible mechanism for the synthesis of 1,2,4-oxadiazolines.

The authors investigated several pyrrolidinyl oxime derivatives both with electron-withdrawing and electron-donating substituents, for the oxidative cyclization to 1,2,4-oxadiazolines with a mechanism as proposed in Scheme 29.

It was observed that triphenylpyrylium (TPP) derivatives were the only effective photocatalysts among those examined, with conversion even in absence of light. As described in the Scheme 29, the reaction begins with the nucleophilic addition to the triphenylpyrylium ion (A) toward intermediate B. Molecular oxygen provides the oxidation of C by regenerates catalyst A. The iminyloxyl radical D undergoes an intramolecular 1,5- hydrogen atom transfer (HAT) to the radical E, which is then oxidized to the iminium ion F. The final 1,2,4-oxadiazoline is generated by intramolecular cyclization of F.

Visible-light-driven photoredox catalysis is attracting interest because of its inherent features of green chemistry and sustainability. In addition to a number of radical reactions, several [2+3] cycloaddition applications have been described in the existing literature (Narayanam and Stephenson, 2011; Nakajima et al., 2016; Staveness et al., 2016; Savateev and Antonietti, 2018).

**Scheme 30:**
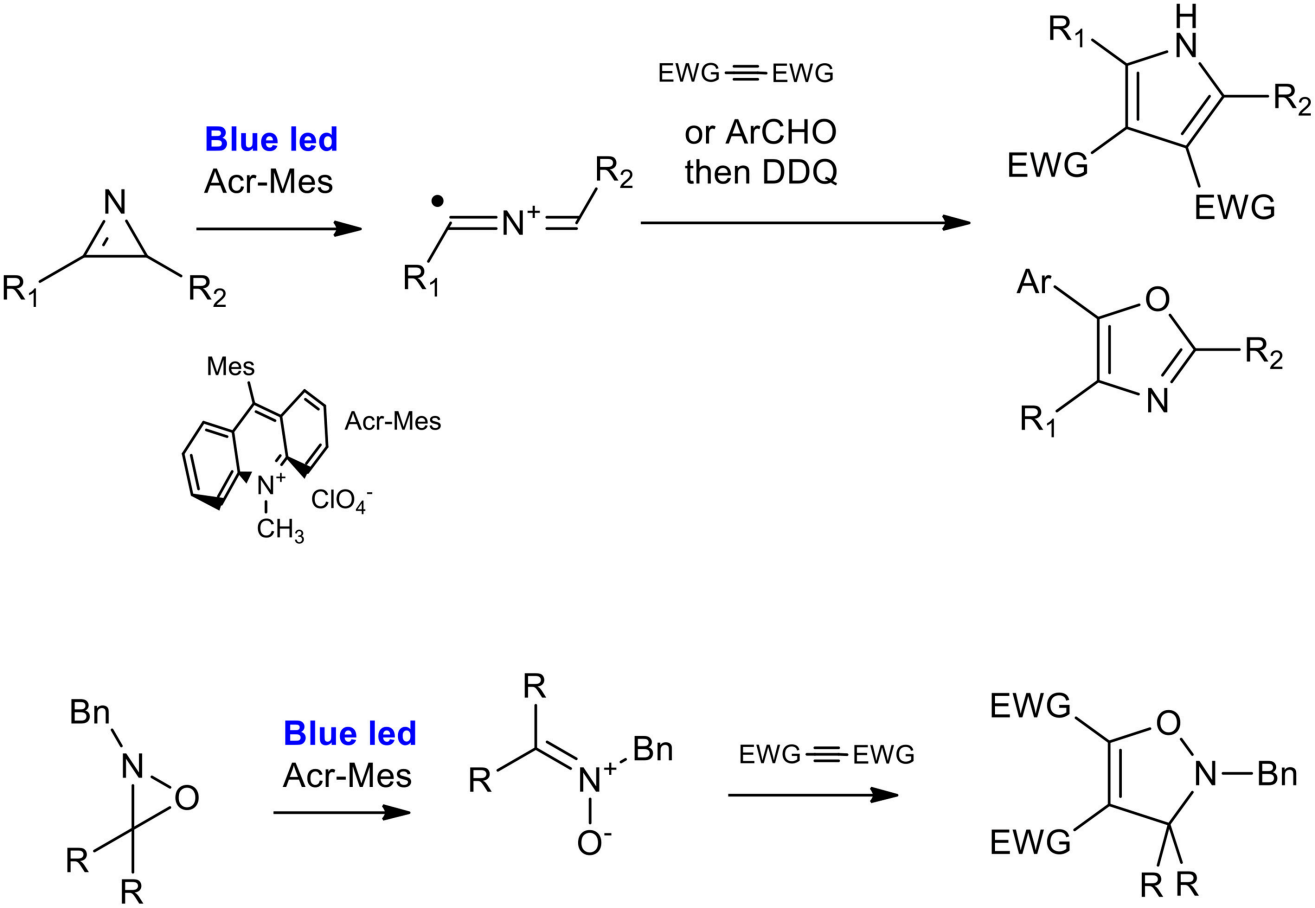
Visible-light mediated synthesis of pyrroles and isozazoles from of *2H*-azirines and *2H*-oxazirine.

2*H*-azirines can react with activated alkynes or aldehydes to produce polysubstituted pyrroles or 2,5-dihydrooxazole, respectively, under very mild reaction conditions (visible-light irradiation, metal-free, and room temperature). As described by Xuan et al., the optimized procedure is catalyzed by 9-mesityl-10-methyl-acridinium perchlorate in dichloroethane under irradiation from a 3 W white LED light. Excellent results were obtained when a range of substituted 2H-azirines were reacted with dimethyl but-2-yne-dioate (Xuan et al., 2014). 2*H*-Azirines reacted with aldehydes in the presence of Li_2_CO_3_. This was done to avoid the oxidation of aldehydes to carboxylic acids, and 2,3-dichloro5,6-dicyano-1,4-benzoquinone (DDQ) was added to the reaction system in order to perform the one pot synthesis of oxazole. The applicability of the reactions was demonstrated using a panel of 12 2,4,5 trisubstituted oxazoles, giving yields in the 40–80% range (Scheme 30) (Zeng et al., 2015).

The ring-opening of 2*H*-oxazirine has been described as occurring via visible-light-mediated photoredox-catalyzed single-electron transfer (SET), giving nitrone precursors. The 1,3-dipolar cycloaddition was therefore used for the synthesis of 4-isoxazolines. 9-mesityl-10-methyl-acridinium perchlorate was the strategic choice of catalyst because of its high oxidizing power (+2.06 V). The [3 + 2] cycloaddition reaction of oxaziridine with dimethyl acetylenedicarboxylate gave very good results when performed in CH_3_CN in the presence of water, used as an additive, and a large set of 4-isoxazolines was synthesized with a good average yield.

The first attempt to describe the mechanism of pyrrole cyclization focused on a radical cycloaddition of the intermediate 2-azaallenyl radical cation, which was in equilibrium with a 1,3-radical-cationic species. Subsequently, the publication mentioned the synthesis of the dihydroisoxazole by an hypothesis of a polar cycloaddition. In fact, the authors proposed the single-electron reduction of the nitrone radical to a nitrone species.

## Author Contributions

All the authors contributed equally to the review preparation. GC supervised the work and edited the final version.

### Conflict of Interest Statement

The authors declare that the research was conducted in the absence of any commercial or financial relationships that could be construed as a potential conflict of interest.
